# The impact of performance‐based financing within local health systems: Evidence from Mozambique

**DOI:** 10.1002/hec.4677

**Published:** 2023-03-27

**Authors:** Laura Anselmi, Julius Ohrnberger, Eleonora Fichera, Pedroso Nhassengo, Quinhas F. Fernandes, Sergio Chicumbe

**Affiliations:** ^1^ Division of Population Health Health Services Research & Primary Care University of Manchester Manchester UK; ^2^ School of Public Health MRC Centre for Global Infectious Disease Analysis Imperial College London London UK; ^3^ Department of Economics University of Bath Bath UK; ^4^ Instituto Nacional de Saúde Maputo Mozambique; ^5^ Department of Global Health University of Washington Washington Seattle USA; ^6^ Direção Nacional de Saúde Publica, Ministério da Saúde Maputo Mozambique

**Keywords:** child and maternal care, difference‐in‐difference, heterogeneity, HIV/AIDS, local health systems, Mozambique, performance based financing

## Abstract

Most evidence on Performance Based Financing (PBF) in low‐income settings has focused on services delivered by providers in targeted health administrations, with limited understanding of how effects on health and care vary within them. We evaluated the population effects of a program implemented in two provinces in Mozambique, focusing on child, maternal and HIV/AIDS care and knowledge. We used a difference‐in‐difference estimation strategy applied to data on mothers from the Demographic Health Surveys, linked to information on their closest health facility. The impact of PBF was limited. HIV testing during antenatal care increased, particularly for women who were wealthier, more educated, or residing in Gaza Province. Knowledge about transmission of HIV from mother‐to‐child, and its prevention, increased, particularly for women who were less wealthy, less educated, or residing in Nampula Province. Exploiting the roll‐out by facility, we found that the effects were concentrated on less wealthy and less educated women, whose closest facility was in the referral network of a PBF facility. Results suggest that HIV testing and knowledge promotion increased in the whole district, as a strategy to boost referral for highly incentivized HIV services delivered in PBF facilities. However, demand‐side constraints may prevent the use of those services.

## 1 INTRODUCTION

Over the last 20 years Performance Based Financing (PBF) has been increasingly implemented in resource‐constrained healthcare systems, as a threefold strategy to widen access, increase quality of care, and ultimately improve population health. Implemented in over 30 low and middle‐income countries (LMICs) (RBF Health, 2020), PBF schemes provide financial incentives to healthcare providers to achieve predefined targets for quantity and/or quality of service delivery (Diaconu et al., 2021; Miller & Babiarz, 2013; Witter et al., 2012). Most schemes in LMICs target measurable indicators of child and maternal care (Kok et al., 2015; Kovacs et al., 2020), including institutional delivery, antenatal care (ANC), and immunization, which are effective ways of improving mothers' and children's health (Benova et al., 2018; Kuhnt & Vollmer, 2017). Fewer schemes target indicators related to other health programmes, for example, HIV/AIDS or nutrition (Kok et al., 2015; Kovacs et al., 2020; Nimpagaritse et al., 2016; Rajkotia et al., 2017; Suthar et al., 2017). Most evaluations showed mixed evidence regarding the average program effects on the delivery of targeted services (Das et al., 2016; Diaconu et al., 2021; Eijkenaar et al., 2013; Mendelson et al., 2017; Miller & Babiarz, 2013; Renmans et al., 2016; Scott et al., 2011, 2016; Witter et al., 2012; Zaresani et al., 2021). There is emerging evidence of heterogeneous effects, which vary by beneficiaries' socio‐economic status (Binyaruka et al., 2018, 2020; Fichera et al., 2021; Van de Poel et al., 2016), and an increasing interest in the mechanisms that may explain the observed differences in the effects (Singh et al., 2021).

Performance Based Financing schemes implemented in LMICs are complex interventions, involving financial incentives, and often also the provision of additional resources, training and supervision (Kok et al., 2015; Kovacs et al., 2020). Those are intended to increase the capacity for delivery, and to improve quality and quantity of the services provided, translating into more widely distributed service use and consequently health benefits. Service use, and therefore the achievement of targets, can be limited by demand‐side constraints, which include financial constraints raised by user fees, travel expenses, time's opportunity costs, or individual health care seeking attitudes (Singh et al., 2021). Heterogenous effects in service use typically arise either because of demand‐side constraints, usually varying by socio‐economic status, or because of differences in service provision. For example, services may differ in type, quality, and mode of delivery, between populations in the immediate catchment area of a PBF facility and those further away served by other facilities.

Local health systems usually comprise health administrations, which oversee and coordinate a network of local providers (Anselmi et al., 2018; Martineau et al., 2018). Health administrations are typically involved in PBF schemes, particularly in supervision, training, and performance verification. They are also rewarded when they achieve targets. Through the response of local health administrations to incentives, PBF may affect not only the services delivered by PBF facilities, but also services' organization, outreach activities, or referrals mechanisms within the local health system (Bertone et al., 2013; Singh et al., 2021; Witter et al., 2013). However, evidence on the effects of PBF beyond enrolled facilities, and their manifestations, is still scarce. With limited exceptions (De Allegri et al., 2018), most evaluations cover schemes rolled out by administrative area, typically districts, and simultaneously in all providers (Kovacs et al., 2020). Evaluators have then defined PBF exposure simply based on whether the schema was rolled‐out in a given health administration (Bonfrer et al., 2014; Fichera et al., 2021; Gage & Bauhoff, 2021; Sherry et al., 2017; Van de Poel et al., 2016). This has prevented the examination of diversified mechanisms of exposure, and related heterogeneous effects, driven by the response of various actors in the system, including health administrations and those providers not directly enrolled in the scheme.

The investigation of heterogenous effects may also be limited by data availability. Many evaluations have used primary data collected in pre‐designated intervention and control areas (Basinga et al., 2011; Binyaruka et al., 2015; Das et al., 2016; Eijkenaar et al., 2013; Falisse et al., 2012; Peabody et al., 2011; Renmans et al., 2016; Soeters et al., 2011; Witter et al., 2012). These include richer information on mechanisms and impact (Anselmi et al., 2017; Bertone et al., 2016; Falisse et al., 2012; Ngo et al., 2016; Peabody et al., 2011), but are limited in temporal and geographical coverage, and generally focus specifically on targeted populations and outcomes. Few studies have used health facility data from national surveys or administrative datasets, with multiple time‐points, to analyze changes in the volume and quality of services delivered (Falisse et al., 2014; Ngo et al., 2016). Heterogeneity in service use or health outcomes within the served population has been studied mostly using data from national household surveys which contain information on health and care outcomes, alongside socio‐economic and other individual and household characteristics (Bonfrer et al., 2014; Fichera et al., 2021; Sherry et al., 2017; Van de Poel et al., 2016).

We analyzed the effects of a PBF scheme targeting 18 healthcare indicators related to the provision of HIV and pre‐ and post‐natal maternal and child health services in Mozambique. The scheme was gradually rolled out across districts, and facilities within districts, in Gaza and Nampula provinces between 2011 and 2017. A previous evaluation assessed the impact of the program on the volume of services delivered by health facilities using routine data up to 2013 from the two intervention and two neighboring provinces (Rajkotia et al., 2017). Most indicators responded differently in each province. At least nine of them, including prevention of mother‐to‐child HIV transmission (PMTCT) and pediatric HIV treatment services, increased by over 50%. The impact was visible after 18 months and was sustained afterward. No negative impact, nor spill‐over effects on non‐incentivized indicators, were detected (Rajkotia et al., 2017). The motivation of healthcare workers also increased (Gergen et al., 2018).

We examined the impact over a longer time‐period of 5 years, and we focused on heterogenous effects on population health care use and knowledge. We used data from the Demographic and Health Surveys (DHS) 2011 and 2015 on the birth of the youngest child, and on mothers' personal and household characteristics, effectively covering births between 2007 and 2015. We linked the DHS data with the national registry of health facilities through clusters' and facilities' geolocation, and we added information on enrollment into PBF for each facility. We exploited the phased roll‐out, and we applied a difference‐in‐difference estimation strategy to assess the effect on indicators which were either directly targeted, or potentially affected by the response to PBF incentives. We focused on the use of maternal and child care services, HIV testing during ANC and knowledge of PMTCT.

This study brings four contributions to the literature on PBF. We add evidence on the population effects of PBF, more specifically on HIV indicators and related knowledge, and on heterogeneities by wealth, education, and province. Finally, using alternative definitions of exposure, we examine effects for populations within and beyond the catchment area of PBF facilities. This allows us to examine the involvement of local health administration and non‐targeted facilities in boosting demand for incentivized services through the district referral network. Testing during antenatal (ANC) increased, particularly for wealthier and more educated women, and for women residing in Gaza. Knowledge of HIV's mother‐to‐child transmission and its prevention increased, particularly for less wealthy and educated women, and for women residing in Nampula. The impact was limited to services provided in the whole district, and it was concentrated in the referral network of PBF facilities. This is where new HIV cases could be found and referred to PBF facilities for highly incentivized and rewarded services. Taken together, the impact on knowledge and on testing beyond the catchment area of PBF facilities, is suggestive of supply‐side attempts to boost demand through HIV/AIDS case finding and behavioral change. The stronger impact on knowledge amongst less wealthy and educated women, and on testing for wealthier and more educated women, indicates that supply‐side attempts to boost demand may be counteracted by socio‐economic constraints to access services.

## 2 PERFORMANCE BASED FINANCING IN MOZAMBIQUE

### 2.1 The healthcare system

Healthcare in Mozambique is mostly publicly funded and provided. There are only few private healthcare facilities and are concentrated in the capital, Maputo City (Instituto National de Saude, 2018). Healthcare is almost free at the point of delivery. Official outpatient fees in public facilities are negligible (MZM 2 to 5 equivalent to USD 0.03–0.08), and exemptions cover the large majority of the population (indigents, children under‐five, pregnant women, chronically ill, patients suffering from malaria, TB and HIV/AIDS) (MISAU, 2012). Primary and secondary healthcare are managed at the district level and in 2012 were provided by 1314 health clinics and health centers and by 66 district hospitals, in 11 provinces and 142 districts, including Maputo City (Figure A1 in Appendix).

Integrated district planning and management have been a key feature of the National Health Service architecture since 1980's, when it was first set up following the independence from Portugal. The core organisational structure remains in place (MISAU, 2002), with an increased number of facilities, more and more complex service provision, and budgets decentralized to the District Governments. National, provincial and district guidelines for planning, management and monitoring recognize the key role of district health systems, including health administrations and health facilities (DPS Cabo Delgado, 2013). Similarly to other settings where the health system is hierarchically organized, the district administrations (Serviço Detrital de Saúde, Mulher e Acção Social, SDSMAS) define and coordinate the role of health facilities within their jurisdiction. They also plan, manage and distribute financial resources, human resources and drugs, and they are responsible for training and supervision (Anselmi et al., 2018; Martineau et al., 2018). All facilities provide basic primary outpatient care. Larger health centers provide full primary care and inpatient care, while district hospitals provide secondary care, including surgery.

Primary care is the backbone of district services. Like in similar settings (Francetic et al., 2020), a hierarchical referral system guarantee the continuum of care. Larger referral facilities, typically provide secondary and tertiary care, serving as a hub for other more peripheral primary care facilities. Each district has one hospital, or a larger health center, which provides secondary care, and is usually located in the major urban center. There are generally one to three referral facilities within a district, depending on its population and geography. These include one district hospital (or larger health center) providing secondary care, and possibly few larger health centers providing primary and inpatient care. The remaining facilities provide basic primary care. The referral system is key to district functioning. Higher and lower‐level facilities within the same referral network collaborate, with lower‐level facilities directing patients to the higher level ones for more complex services, and serving as a base for outreach activities organized by the higher‐level facilities. Coordinated and supported by district health administrations, higher‐level facilities oversee planning and supervision within lower‐level facilities, and organize various outreach activities, mostly for preventive services. Additionally, there are voluntary health workers supporting health facilities, particularly smaller health centers, through the provision of outreach services in the community.

Maternal and child health services are provided in all facilities. However, some clinics lack the resources to assist delivery, which tend to happen in larger health centers or district hospitals (MISAU, 2012). HIV antiretroviral treatment (ART) is provided only in few designated facilities within a district, typically the larger ones. Rapid HIV testing can be delivered in all facilities and through outreach campaigns. Despite improvements in service delivery and access, over the last decade vaccinations and institutional deliveries appeared to have decreased between 2011 and 2015 (MISAU et al. (2015), likewise resources from international donors (Anselmi, L 2017).

### 2.2 Performance Based Financing

The implementation of Performance Based Financing started in January 2011 in two of 11 provinces with different population and health characteristics. Gaza, in the South, was amongst the provinces with the highest HIV prevalence (about 25.1%), and Nampula, in the North, was amongst those with the lowest (of 4.6%) (MISAU, 2009). In Gaza only 18% of the population was in the three lowest wealth quintiles, compared with 62% in Nampula (Table 2).

The PBF program was funded by the United States President's Emergency Plan for AIDS Relief (PEPFAR) through the Center for Disease Control (CDC) and was implemented by the Elizabeth Glaser Pediatric AIDS Foundation (EGPAF). The CDC was already actively supporting Gaza and Nampula provinces in providing HIV/AIDS care. The health facilities enrolled in the program were selected from those serving a large population and offering HIV/AIDS services, including adult or pediatric ART and PMTCT. Those facilities had to meet minimum staffing requirements and, either have a bank account, or be under the financial supervision of the SDSMAS. The PBF scheme was rolled‐out over four periods: Phase 1 starting in January 2011 (30 facilities in Nampula and in 18 Gaza); Phase 2 in March 2012 (41 facilities in Nampula and in 23 Gaza), Phase 3 in September 2013 (15 facilities in Nampula and 25 in Gaza) and Phase 4 in September 2014 (no facilities in Nampula and 21 in Gaza). No new districts were enrolled from September 2013 onwards (Figures A2 and A3 in Appendix). No change was implemented in the remaining facilities as part of the scheme.

The PBF scheme was a complex intervention which: provided incentives to improve the delivery of specific services; increased resources available; strengthened capacity; and affected local governance. The scheme incentivized 21 facility indicators, listed in Table 1 with the relative prices, which were mostly taken from the existing monitoring frameworks, and which covered maternal and child care, tuberculosis, PMTCT, pediatric HIV, adult HIV care and treatment (Rajkotia et al., 2017). Additional incentivized indicators related to the monitoring and management functions of the SDSMAS, included: health facilities supervision, district PBF committee meetings, timely submission of correct monitoring reports, efficient management of human resources, financial management aligned with set norms and procedures, assessment of health facilities quality (DPS Nampula, 2018). Some indicators were changed when targets were reached, but this happened after the period covered by this study. Performance was assessed quarterly, and jointly by EGPAF and the Provincial Directorate of Health (Gergen et al., 2018). Quality was assessed every 6 months, with check lists covering prevention and control of diseases, and maternity and HIV services (Gergen et al., 2018). Incentives consisted of a quantity‐based bonus, with weighting for quality and remoteness, which could be distributed to staff as salary top‐up (60% share) or used to improve facilities (40% share). Bonuses contributed up to 50% of the operating costs (Gergen et al., 2018). Targeted districts received also additional resources and training.

**TABLE 1:** Summary of targeted indicators and related outcomes in DHS 2011 and 2015.

Indicators per area	Price Gaza (USD)	Price Nampula (USD)	Indicators measured from DHS data
Maternal and Child Health			
1. Four antenatal care consultations (ANC)	2	2	Number of ANC, 4 ANC, ANC with professional
2. Facility deliveries	3	3	Institutional delivery, institutional delivery with professional
3. Complete vaccination (9–12 months)	1.8	1.8	Full immunization at 9 and at 12 months
4. Family planning general	0.1	0.1	
5. Post‐partum consultation (3–28 days)	1.6	1.6	
6. Nutritional program ‐ Children with acute malnutrition who completed treatment and satisfied the defined clinical criteria for discharge	1.2	1.5	
Pediatric HIV			
1. Children 0–14 months tested for HIV		4.2	
2. Children 0–23 months who initiated ART	7.7	7.7	
3. Children 2–14 years who initiated ART	7	7	
4. Children 0–14 alive on ART 12 months after initiating	11.2	11.2	
TB			
1. Children diagnosed with TB who started the treatment			
2. HIV+ adults who started TB treatment	2.8	2.8	
Adult HIV			
1. Adults who initiated ART	4	4	
2. HIV+ patients 6 months on Isoniazid to prevent TB	2	2	
3. Adults alive on ART 12 months after initiating	8	8	
4. Patients lost to follow up who return for councelling and treatment			
Prevention of HIV transmission from mother to child			Mother's knowledge of mother to child transmission and knowledge of drugs for preventing transmission
1. Pregnant HIV+ women on ART 6 m to prevent transmission	6.25	6.25	Mother offered test or tested at ANC consultation
2. Pregnant women who initiated ART	10	10	
3. Family planning for HIV+ women	5	5	
4. PCR test for children 4–8 weeks	4.9	4.9	
5. Children, born to HIV+ women, who were tested via rapid test 9–12 months after birth	4.2		
6. HIV rapid test definitively negative for children 9–18 months			
7. Partners tested			

### 2.3 Rationale for heterogeneous effects by exposure

With a roll‐out by facility, changes stimulated by the incentives could affect the population in a district in different ways, depending on the response of the PBF facilities, of the health administration, and of the remaining facilities (Bertone et al., 2013). Testing the impact of the scheme for different populations, can therefore provide insights on the involvement of health administrations and facilities in the response to PBF incentives.

First, the whole district population may be affected because of the overarching activity of health administrations. Providing health administrations with additional funding, and rewarding them for their own and their health facilities' performance, may stimulate improvements in their functions. These can in turn lead to improved resources, supervision, training, and coordination in all facilities, as well as increased vaccinations, information campaigns, and outreach activities. Ultimately, we may expect improved service delivery, and increased use by the served population, particularly for targeted services, or those complementary to them. For example, we may expect increased information campaigns or HIV testing, to find HIV cases and stimulate demand for the highly rewarded ART services.

Second, improved provision of targeted services in PBF facilities can affect all population groups within a reachable distance, either by satisfying previously unmet demand, or by stimulating further demand through increased quality. Patients directly served by a PBF facility, typically those for whom that is the closest facility, are the most likely affected. However, while it would be natural for patients to seek care from their closest facility, some may bypass the referral system and seek care from facilities which are further away, but offer more comprehensive or higher quality services (Leonard, 2014). The improved reputation of PBF facilities, or the implementation of outreach activities beyond their immediate catchment area to attract patients, may stimulate bypassing. Bypassing is more likely to involve patients with lower demand‐side constraints (e.g., those with lower opportunity cost of traveling further away, or those with higher perceived benefit from better care) (Singh et al., 2021).

Third, 26% of PBF facilities are referral facilities (73% in phase1, 29% in phase 2, 5% in phase 3% and 0% in phase 4). Referral facilities attract and receive patients from the catchment areas of the peripheral facilities in their network. With the support of health administrations, they also organize outreach activities, share good practices, and provide training and resources to more peripheral facilities (Give et al., 2019). These activities may intensify if PBF referral facilities actively seek to boost demand and reach the target for incentivized services, by increasing the referral of patients from the catchment areas of peripheral facilities (Singh et al., 2021). Populations in the referral network of a PBF facility, but in the catchment area of a non‐PBF facility, can therefore be affected by the scheme.

## 3 DATA

### 3.1 Demographic and Health Surveys

We used two waves of the Demographic Health Survey (DHS) Mozambique, DHS 2011 and AIS 2015 (MISAU, 2011, 2018). The DHS Program provides researchers with accurate and representative data for over 90 countries. These data are of the highest standard achievable in Mozambique and are used as national and international reference to monitor the progress of child and maternal health indicators. DHS surveys have been previously used for robust impact evaluations elsewhere (Bonfrer et al., 2014; Fichera et al., 2021; Gage & Bauhoff, 2021; Sherry et al., 2017; Van de Poel et al., 2016).

The DHS is a repeated cross‐sectional survey, nationally representative of women in reproductive age (15–49), of their children, and of men aged 15–49 (or 59). The household questionnaire covers the roster (age sex, relationship to the head of the household, education, parental survivorship, residence, and birth registration) and various other characteristics. Household characteristics include the information used to compute the DHS wealth index, namely: asset ownership, materials used for housing construction, and access to water and sanitation (Rutstein & Johnson, 2004). The individual questionnaire cover fertility, mortality, family planning, marriage, reproductive health, child health, nutrition, and HIV/AIDS. The geographic coordinates of the cluster, village or urban neighborhood of residence at the time of the interview, are made available with a random displacement of maximum 5 Km (DHS, 2020). Women of reproductive age, who have been pregnant during the 5 years preceding the interview, are asked about their use of care prior to, during, and post‐delivery, and about the care, survival, and health of their children. Information on child mortality and vaccination history is collected for all births in the 5 years preceding the interview, while information on ANC, institutional delivery and post‐natal care is collected only for the last birth. All information is reported by the mother.

We used information on child vaccination, and on mother's healthcare use and knowledge about HIV PMTCT. We considered the most recent birth only, and we constructed a pooled cross‐sectional sample of births, and related mothers and children, covering the period between 2007 and 2015. We restricted the sample to Gaza, Nampula and their neighboring provinces Inhambane, Maputo Province and Zambezia, which exhibited parallel trends pre‐intervention for the majority of outcomes. We used 3937 observations for the analysis of HIV knowledge and testing, ANC and institutional delivery, and 2759 observations (excluding those born less than 12 months prior to the interview) for immunization within 9 or 12 months.

### 3.2 Service Availability and Readiness Assessment (SARA) survey

We created a census of health facilities operating between 2007 and 2015 using data on the exact geo‐location, type of facility and opening year from the 2018 Service Availability and Readiness Assessment (SARA) Survey (Instituto National de Saude, 2018). The SARA includes information on various characteristics (e.g., location, opening year, type, access to water and electricity) and resources (e.g., staff, equipment and drugs availability) for every health facility, except for six located in remote islands which were not reachable at the time of the survey. Unfortunately, the SARA does not indicate the services provided by each facility, for example, HIV/AIDS anti‐retroviral treatment. We checked for facility closure using a previous census validated in 2011 (Anselmi et al., 2015). No facilities were opened or closed in any PBF district between the roll‐out of the scheme in 2011 and the end of the study period in 2015. We identified each facility's referral facility using information about districts from national and provincial operational plans and data on the volume of services delivered by each facility from the national health information system (Anselmi et al., 2018). We validated our list with the Ministry of Health.

### 3.3 Performance based financing roll‐out

The EGPAF provided information on the year and month in which each district and each facility were enrolled in PBF and started receiving bonus payment (Figures A2 and A3 in Appendix). We considered a SDSMAS enrolled when PBF was rolled‐out in at least one facility in the district.

### 3.4 Outcomes

For each birth, we considered 11 of the available outcomes that can be affected by changes in the availability and quality of targeted services, and which are either directly targeted by PBF, or functional to increasing their demand (Table 1).

#### 3.4.1 Antenatal care and HIV testing

We used five measures of ANC use and quality, including a continuous measure of the total number of ANC visits and a set of binary indicators taking value one if the mother: (i) had at least four ANC visit; (ii) attended ANC with a qualified care professional; (iii) was offered an HIV test; (iv) was tested during ANC visits. Although testing was not directly incentivized, we hypothesized an effect because a test is required to identify potential users of incentivized HIV services. The administration of ART to HIV+ pregnant women to prevent transmission (PMTCT) (6.5 USD), and the initiation of antiretroviral treatment (ART) by pregnant women (USD 10) are two highly rewarded indicators. Their delivery requires not only identifying target mothers throuh testing, but also sensitising them to increase demand for treatment and adherence. Testing can be performed both in PBF and non‐PBF facilities, during ANC consultations in a facility, or through outreach activities.

#### 3.4.2 Knowledge about transmission of HIV from mother‐to‐child

We used two binary indicators. The first takes value one if the mother was aware of HIV and of its transmission from mother‐to‐child (MTC) during pregnancy, delivery, and via breastfeeding. The second takes value one if the mother was aware of drugs that can prevent mother‐to‐child transmission (PMTCT). Those two variables are proxies for the intensity and quality of activities aimed at HIV prevention, which when effective can increase awareness. Because PBF targeted the provision of complete PMTCT, we hypothesized an increase in HIV awareness activities, both in PBF and non PBF facilities, or in the community, which would increase knowledge.

#### 3.4.3 Institutional delivery

We used two binary indicators for institutional delivery, the first taking value one if the most recent birth took place in a healthcare facility, and the second taking value one if the delivery was assisted by a professional.

#### 3.4.4 Immunization

We generated two binary variables taking value one if the child was fully vaccinated either within 9 months, as per international guidelines, or within 12 months, as per Mozambique guidelines. The full vaccination cycle included polio (three doses), tuberculosis (*Bacillus* Calmette–Guérin, BCG), diphtheria, pertussis (whooping cough), and tetanus (DPT) (three doses). Using the standardized codes provided by the DHS program, we could generate the variables only for children with a vaccination card and with valid vaccination dates reported. Vaccinations are administered through routine outpatient visits or through outreach campaigns organized by provincial and district health administrations.

### 3.5 Control variables

We included a set of covariates measured at the time of the interview. We included years of education, age (15–49) in 5 years bins, and a binary indicator for Christian religion of the mother. Not only behaviors may differ by religion, but Christian faith organizations are involved in providing child and maternal healthcare with the potential of affecting outcomes more favorably in this group (Widmer et al., 2011). We controlled for household composition, including: (i) whether the head of the household was female, and their age; (ii) the number of children of age five and below, to account for experience in childcare; and (iii) the number of household members, to account for resource generation and use. We accounted for household wealth, measured by the DHS wealth index in quintiles (Rutstein & Johnson, 2004), for urban versus rural residence, and for access to services. We proxied access to services by the distance to the nearest health facility (crow fly distance in Km between DHS cluster and facility geolocation), and by car or truck ownership.

## 4 METHODS

### 4.1 Average effects

In line with similar evaluations (Bonfrer et al., 2014; Fichera et al., 2021; Gage & Bauhoff, 2021; Sherry et al., 2017; Van de Poel et al., 2016), we defined exposure as residence in a PBF district, at the time of conception (9 months prior to the date of birth), assuming that the mother had not moved between then and the interview.

We estimated the effect of PBF on the outcomes of interest, using a difference‐in‐difference (DID) estimation strategy, which compares changes in exposed (treated) and unexposed (control) households, before and after the implementation of PBF (Ashenfelter, 1978; Bonfrer et al., 2014; Fichera et al., 2021; Van de Poel et al., 2016).

We estimated Equation (1) separately for each outcome:

(1) $$Y_{itl}=\beta_{0}+\beta_{1}{DID}_{itl}+\beta_{2}Post_{t}+\beta_{3}X_{itl}+\delta_{t}+\lambda_{f}+\epsilon_{itl}$$

where $Y_{itl}$ is the outcome for mother (or child) *i*, in area *l* at time *t*. ${DID}_{itl}$ varies due to the phased roll‐out, and takes value one if conception happened after PBF was implemented in area *l*, and zero otherwise. $\beta_{1}$ is the coefficient of interest, indicating the effect of PBF on mother's (or child's) outcome.

$Post_{t}$ is a set of four binary variables (three when exposure is defined by district as only new facilities were enrolled in phase four), indicating if child conception was before or after the start of each roll‐out phase. $X_{itl}$ is a vector of control variables, including household's and mother's characteristics, distance and squared distance from the closest facility, accounting for linear and non‐linear effects, and survey's year. $\delta_{t}$ are two sets of time fixed effects for year and month of conception, controlling for unobserved time‐varying heterogeneity, for example, in access to services. $\lambda_{f}$ are district fixed effects and $\epsilon_{itl}$ is the individual idiosyncratic error term.

We estimated Equation (1) using linear probability models, with robust standard errors clustered at the DHS primary sampling unit (cluster) and with women's individual population weights (Croft et al., 2018). We accounted for multiple hypothesis testing (11 outcomes) using the Bonferroni‐Holm method to estimate family‐wise *p*‐values using 500 bootstraps. (Giacalone et al., 2018; Holm, 1979; Jones et al., 2019).

### 4.2 Heterogenous effects by wealth, education, and province

Heterogenous outcomes by wealth, education, or contextual characteristics, may reflect differences in the opportunity cost of seeking or using care, and highlight the presence of demand‐side constraints (Binyaruka et al., 2018, 2020; Fichera et al., 2021; Van de Poel et al., 2016).

We replicated the analysis for each subgroup separately, as in similar recent studies (Fichera et al., 2021). We characterized sub‐groups by mother's wealth (three lower vs. two higher quintiles), and education (up to four vs. five or more), with thresholds approximately defined by the median. We then tested heterogenous effects by province. We re‐estimated Equation (1) on sub‐groups including each treated province and the relative control group, first Nampula with Zambezia, and then Gaza with Maputo Province and Inhambane.

### 4.3 Heterogeneous effects by exposure

We investigated response mechanisms within the district, by considering three alternative definitions of exposure to PBF, depending on the hypothesized involvement of health administration and non‐PBF facilities. We re‐estimated Equation (1) for each outcome, with each alternative definitions of exposure, and with facility rather than district fixed effects.

First, we considered clusters (villages) exposed, if PBF was implemented in the closest health facility, where the population would naturally first seek routine care, or from which it would receive any outreach service. A significant effect associated with this, but not other definitions of exposure, would suggest a poor response by health administrations and other facilities in the referral network, with benefits restricted to the immediate catchment area of PBF facilities.

Second, we considered that populations could seek care within a reachable distance, and we defined as exposed those clusters within a given distance from a PBF facility. We defined “reachable distance” based on the radius of the designated catchment area, which varies across facilities, and is generally determined by district planners so that each village is in the catchment area of at least one facility. The radius is larger where the population is sparser, and varies between 8 and 18 Km outside the capital. We used the radius in Gaza (15 Km), which is larger than in Nampula (11 Km) (MISAU, 2012). For each cluster, the closest facility, and possibly also others, were situated within a distance of 15 Km. A significant effect associated with this, but not other definitions of exposure, could indicate that PBF facilities sought to stimulate demand beyond their usual catchment area, inducing patients to bypass the referral system.

Finally, we considered that PBF could trigger a supply response within the whole referral network, and we defined a cluster exposed if its closest facility was in the referral network of a PBF facility. The referral network has a geographical coverage which in some cases is equivalent to the whole district. Therefore exposure based on the referral network overlaps only partially with exposure based on distance from a PBF facility. A significant effect associated with this, but not other definitions of exposure, would suggest that PBF facilities exploited their network to increase demand and referral for targeted services.

### 4.4 Parallel trends

DID estimation for causal inference requires the assumption of parallel trends in the outcome variables between treated and non‐treated units in the pre‐intervention period (Angrist & Pischke, 2008). For each outcome, we tested for both linear and non‐linear parallel trends by re‐estimating Equation (1) on observations related to conceptions which happened before the roll‐out of PBF (January 2011). First, we introduced the interaction between future PBF exposure and conception‐date linear time trends. Second, we interacted bi‐annual 6‐months periods with future exposure to PBF to test for difference between exposed and not exposed in each time period. We graphed the coefficient estimates with their 95% confidence bands, and we considered parallel trends to hold if the estimates were not significantly different from zero (Wichman, 2017). We tested the parallel trends assumption for each sub‐group used for the analysis of heterogeneous effects, as well as for the robustness checks described in the next section.

### 4.5 Robustness checks

We run several robustness checks. First, we re‐estimated the models excluding all mothers living in non‐PBF districts in Nampula and Gaza, to remove potential bias within province from the involvement of the Provincial Directorate of Health. Second, we re‐estimated the models for heterogeneous effects associated with exposure defined either by distance or by referral facility's enrolment by excluding mothers residing in PBF districts, but not directly exposed, to avoid bias from potential effects within the districts. Third, we re‐estimated the regressions for binary outcomes using non‐linear models. Fourth, we tested for potential selection of districts into the PBF program using district‐level data on health facilities, population health, socio‐economic, and other characteristics available for the pre‐intervention period (Anselmi et al., 2018). We estimated the association between PBF roll‐out and district characteristics with two logistic models, using either information for 2010 or the average between 2008, 2009 and 2010. Finally we replicated the analysis for exposure by distance using a radium of 25 Km, a round‐up of the distance walkable in the maximum time distance to a facility reported in national household surveys (4 h), at a human walking speed of 6 Km/hour (MISAU, 2012).

## 5 RESULTS

### 5.1 Descriptive statistics

Table 2 presents the descriptive statistics separately for Gaza, Nampula, and the control group, which includes Maputo Province, Inhambane e Zambezia, before and after the implementation of PBF. The exposure of mothers to PBF varied in Gaza between 68%, when defined by district, and 26%, when defined by closest facility, and in Nampula between 64% and 21%.

**TABLE 2:** Descriptive statistics by Gaza, Nampula and control provinces, means pre and post 2011.

	Pre 2011			Post 2011		
	Gaza	Nampula	Control	Gaza	Nampula	Control
Exposure to PBF						
At least one HF with PBF in the district	0.00	0.00	0.00	0.68	0.64	0.00
Closest HF with PBF	0.00	0.00	0.00	0.26	0.21	0.00
HF within 15 Km with PBF	0.00	0.00	0.00	0.41	0.36	0.00
HF within 25 Km with PBF	0.00	0.00	0.00	0.45	0.38	0.00
Referral of closest HF with PBF	0.00	0.00	0.00	0.68	0.64	0.00
Outcome						
ANC visits (number)	4.09	3.12	3.54	4.55	3.04	3.59
Four ANC visits	0.66	0.39	0.53	0.74	0.38	0.55
ANC with professional	0.97	0.93	0.86	0.99	0.91	0.86
Offered HIV test at ANC	0.75	0.46	0.66	0.88	0.61	0.68
Tested for HIV at ANC	0.70	0.39	0.66	0.86	0.60	0.68
Knowledge of HIV vertical transmission	0.63	0.67	0.78	0.63	0.58	0.65
Knowledge of HIV vert. trans. prevention	0.71	0.56	0.79	0.82	0.61	0.68
Institutional delivery	0.74	0.59	0.60	0.81	0.61	0.62
Institutional delivery with professional	0.72	0.59	0.55	0.80	0.60	0.61
Fully vaccinated at 12 months	0.67	0.52	0.60	0.41	0.22	0.28
Fully vaccinated at 9 months	0.40	0.27	0.37	0.28	0.13	0.20
Observations	453	388	1376	354	411	955
Observations (full vaccination)	346	271	912	295	269	666

Controls	Pre 2011				Post 2011		
	Gaza	Nampula	Control	Gaza		Nampula	Control
Distance from closest HF (km)	3.05	5.27	5.17	4.09		5.17	5.32
	(2.15)	(3.73)	(4.58)	(2.95)		(3.57)	(4.88)
Survey 2011	1.00	1.00	1.00	0.27		0.31	0.40
Survey 2015	0.00	0.00	0.00	0.73		0.69	0.60
Birth month: Jan	0.09	0.07	0.07	0.10		0.11	0.09
Birth month: Feb	0.08	0.06	0.07	0.09		0.10	0.10
Birth month: Mar	0.08	0.09	0.09	0.12		0.12	0.10
Birth month: Apr	0.07	0.11	0.09	0.12		0.12	0.12
Birth month: May	0.07	0.09	0.08	0.12		0.09	0.14
Birth month: Jun	0.07	0.10	0.09	0.10		0.13	0.12
Birth month: Jul	0.07	0.09	0.09	0.06		0.07	0.09
Birth month: Aug	0.10	0.08	0.09	0.05		0.07	0.08
Birth month: Sep	0.12	0.09	0.10	0.08		0.06	0.07
Birth month: Oct	0.08	0.06	0.09	0.06		0.04	0.03
Birth month: Nov	0.09	0.07	0.07	0.05		0.05	0.03
Birth month: Dec	0.08	0.08	0.08	0.05		0.05	0.04
Birth year: 2006	0.00	0.00	0.00	0.00		0.00	0.00
Birth year: 2007	0.00	0.01	0.01	0.00		0.00	0.00
Birth year: 2008	0.21	0.19	0.19	0.00		0.00	0.00
Birth year: 2009	0.34	0.36	0.31	0.00		0.00	0.00
Birth year: 2010	0.44	0.45	0.49	0.00		0.00	0.00
Birth year: 2011	0.00	0.00	0.00	0.27		0.31	0.40
Birth year: 2012	0.00	0.00	0.00	0.00		0.00	0.00
Birth year: 2013	0.00	0.00	0.00	0.26		0.24	0.20
Birth year: 2014	0.00	0.00	0.00	0.31		0.30	0.25
Household size (number)	6.65	4.96	5.86	6.91		5.32	6.11
	(3.16)	(1.74)	(2.55)	(3.32)		(1.92)	(2.80)
15–19 years	0.11	0.10	0.10	0.21		0.18	0.18
20–24 years	0.26	0.32	0.22	0.27		0.25	0.29
25–29 years	0.23	0.19	0.24	0.18		0.20	0.20
30–34 years	0.20	0.18	0.20	0.18		0.18	0.14
35–39 years	0.13	0.14	0.14	0.12		0.11	0.12
40–44 years	0.06	0.05	0.07	0.05		0.06	0.05
45–49 years	0.01	0.02	0.03	0.01		0.02	0.02
Children in household (number)	1.82	1.60	1.64	2.06		1.78	1.86
	(1.04)	(0.72)	(0.90)	(1.14)		(0.80)	(0.92)
Female household head	0.55	0.31	0.35	0.54		0.26	0.34
Age of household head (years)	41.75	34.37	38.22	40.82		33.40	37.95
	(15.45)	(11.33)	(12.92)	(14.17)		(10.43)	(13.69)
Wealth quintile 1	0.02	0.32	0.20	0.03		0.35	0.23
Wealth quintile 2	0.04	0.26	0.17	0.04		0.25	0.15
Wealth quintile 3	0.15	0.20	0.14	0.20		0.20	0.16
Wealth quintile 4	0.57	0.14	0.22	0.48		0.13	0.25
Wealth quintile 5	0.22	0.09	0.27	0.25		0.08	0.22
Years in education (number)	4.23	2.99	3.76	4.88		3.10	4.49
	(3.34)	(3.14)	(3.37)	(3.32)		(3.18)	(3.55)
Christian religion	0.66	0.48	0.54	0.74		0.49	0.61
Rural residence	0.76	0.64	0.67	0.72		0.72	0.66
Car ownership	0.07	0.01	0.05	0.10		0.01	0.05
Observations	453	388	1376	354		411	955

*Note*: All variables are binary and means are unweighted. (SD) reported for non‐binary variable only.

Outcomes were better in Gaza than in Nampula before 2011. The average number of ANC visits per pregnant woman was 4.1 in Gaza and 3.1 in Nampula, and the percentage of mothers with at least four ANC was 66% in Gaza and 39% in Nampula, with most consultations done with a professional in both Provinces. The percentage of mothers offered HIV‐tests and tested for HIV at ANC were 75% and 70% in Gaza and 46% and 39% in Nampula. Complete knowledge of HIV mother‐to child transmission and knowledge of existing drugs to avoid transmission were 63% and 71% in Gaza and 67% and 56% in Nampula. Institutional deliveries were higher in Gaza (74%) than in Nampula (59%), and they were mostly attended by a professional. Sixty‐seven percent of children in Gaza and 52% in Nampula had a vaccination card reporting each vaccination date, and were fully vaccinated within 12 months, according to the full vaccination cycle duration in Mozambique. Most outcomes tended to improve, except for vaccinations, ANC visits and knowledge of HIV vertical transmission, in Nampula. Table A1 in Appendix presents outcomes in Gaza and Nampula, before and after the implementation of PBF, and for exposed and not exposed samples.

There was variation in the control variables between the two PBF provinces and the control group. Seventy‐nine percent of mothers were in the two richest quintiles in Gaza, versus only 21% in Nampula and 47% in the control group. Education was higher in Gaza, 4.3 years, compared with 3.0 years in Nampula, and 3.8 years in the control group. Distance from the closest health facility was lower in Gaza (3.1 Km) than in Nampula (5.3 Km) and in the control group (5.2 Km). Most mothers lived in rural areas: 76% in Gaza, 64% in Nampula and 54% in the control group. There were also demographic differences, for example, in the age of the mother, in the average number of children under 5 per household, in the average household size and in the gender and age of the head of household.

### 5.2 Average effect

Table 3 presents the effect of PBF on each outcome. Performance Based Financing had a positive effect only on HIV test performed during ANC (13 pp, Bonferroni‐Holm corrected *p* = 0.037), on knowledge of HIV mother to child transmission (MTCT) (19 pp, *p* < 0.001) and on knowledge of drugs to prevent it (PMTCT) (31 pp, *p* < 0.001). There were no significant effects on any other outcome.

**TABLE 3:** Average effects of Performance Based Financing.

	Number of ANC visits	Four ANC visits	ANC with professional	Offered HIV test at ANC	Tested for HIV at ANC	Knowledge of HIV vertical transmission	Knowl. of HIV vert. transm. prevention	Institutional delivery	Institutional delivery with professional	Fully vaccinated 12 months	Fully vaccinated 9 months
PBF effect	−0.134	−0.007	−0.042	0.089	0.134**	0.193***	0.310***	−0.027	−0.058	−0.059	−0.060
	(0.177)	(0.049)	(0.031)	(0.046)	(0.046)	(0.047)	(0.038)	(0.045)	(0.045)	(0.040)	(0.036)
R‐sq	0.242	0.148	0.276	0.310	0.336	0.132	0.182	0.335	0.338	0.321	0.184
Linear parallel trends	Yes	Yes	Yes	Yes	Yes	No	Yes	Yes	Yes	Yes	No
Non‐linear parallel trends	No	No	No	Yes	Yes	No	No	No	No	Yes	Yes
Observations	3937	3937	3937	3937	3937	3937	3937	3937	3937	2759	2759

*Note*: Constant, year of birth, month of birth, distance to health facility, squared distance to health facility, mothers' wealth quintile, years of education, 5‐year age‐group, number of children, Christian religion, household size, female head of household, age of head of household, rural residence, car ownership and fixed effects for closest health facility. DHS population weights applied to observations. Standard errors clustered at the DHS primary sampling unit level in parentheses.

*p < 0.10, **p < 0.05, ***p < 0.01, based on Bonferroni‐Holm *p*‐value.

### 5.3 Heterogenous effects by wealth, education and province

Table 4 presents the effects by wealth, education and province. The increase in HIV testing was more pronounced for wealthier women (19 pp, *p* = 0.001), more educated women (13 pp, *p* = 0.096), and in Gaza (17 pp, *p* = 0.003). Conversely the increase in HIV related knowledge was higher for less wealthy women (28 pp, *p* < 0.001 for MTCT and 38 pp, *p* < 0.001 for PMTCT), for less educated women (29 pp, *p* < 0.001 for MTCT and 43 pp, *p* < 0.001 for PMTCT) and in Nampula (30 pp, *p* < 0.001 for MTCT and 37 pp, *p* < 0.001 for PMTCT).

**TABLE 4:** Effects of performance based financing by wealth, education and province.

	Number of ANC visits	Four ANC visits	ANC with professional	Offered HIV test at ANC	Tested for HIV at ANC	Knowledge of HIV vertical transmission	Knowl. of HIV vert. transm. prevention	Institutional delivery	Institutional delivery with professional	Fully vaccinated 12 months	Fully vaccinated 9 months
Wealth											
Below median	−0.198	−0.007	−0.1	0.082	0.100	0.279***	0.380***	−0.004	−0.050	−0.033	−0.049
	(0.249)	(0.064)	(0.048)	(0.069)	(0.067)	(0.062)	(0.052)	(0.063)	(0.064)	(0.051)	(0.046)
R‐sq	0.220	0.137	0.283	0.235	0.249	0.177	0.161	0.247	0.253	0.271	0.167
Linear parallel trends	Yes	Yes	Yes	Yes	No	No	Yes	Yes	Yes	Yes	No
Non‐linear parallel trends	No	No	No	Yes	Yes	No	Yes	Yes	Yes	No	No
Observations	2020	2020	2020	2020	2020	2020	2020	2020	2020	1293	1293
Above median	0.057	0.018	0.053	0.118	0.192**	0.080	0.215***	−0.051	−0.072	−0.049	−0.025
	(0.194)	(0.069)	(0.024)	(0.048)	(0.049)	(0.060)	(0.050)	(0.043)	(0.043)	(0.054)	(0.057)
R‐sq	0.201	0.156	0.191	0.229	0.259	0.118	0.166	0.202	0.188	0.434	0.254
Linear parallel trends	Yes	Yes	Yes	Yes	Yes	Yes	Yes	Yes	Yes	Yes	Yes
Non‐linear parallel trends	No	No	No	Yes	Yes	Yes	Yes	Yes	Yes	Yes	Yes
Observations	1917	1917	1917	1917	1917	1917	1917	1917	1917	1466	1466
Education											
0–4 years	−0.135	−0.041	−0.028	0.098	0.126	0.292***	0.428***	0.013	−0.021	−0.052	−0.093*
	(0.230)	(0.060)	(0.043)	(0.066)	(0.066)	(0.065)	(0.053)	(0.061)	(0.060)	(0.054)	(0.046)
R‐sq	0.249	0.152	0.302	0.282	0.292	0.141	0.169	0.267	0.275	0.280	0.176
Linear parallel trends	Yes	Yes	Yes	Yes	Yes	No	No	Yes	Yes	Yes	No
Non‐linear parallel trends	Yes	Yes	Yes	Yes	Yes	Yes	Yes	Yes	Yes	Yes	Yes
Observations	2241	2241	2241	2241	2241	2241	2241	2241	2241	1486	1486
4 years or more	−0.060	0.012	0.002	0.077	0.135*	0.024	0.112	−0.073	−0.094	−0.094	0.011
	(0.200)	(0.071)	(0.026)	(0.053)	(0.051)	(0.055)	(0.051)	(0.051)	(0.051)	(0.057)	(0.055)
R‐sq	0.207	0.160	0.169	0.250	0.268	0.199	0.201	0.322	0.314	0.464	0.291
Linear parallel trends	Yes	yes	Yes	Yes	Yes	Yes	Yes	Yes	Yes	Yes	Yes
Non‐linear parallel trends	Yes	Yes	Yes	Yes	Yes	Yes	Yes	Yes	Yes	Yes	No
Observations	1696	1696	1696	1696	1696	1696	1696	1696	1696	1273	1273
Province											
Nampula	−0.252	−0.043	−0.082	0.058	0.088	0.300***	0.367***	−0.049	−0.082	−0.103	−0.083
	(0.239)	(0.068)	(0.048)	(0.068)	(0.067)	(0.057)	(0.050)	(0.058)	(0.058)	(0.061)	(0.049)
R‐sq	0.220	0.137	0.273	0.242	0.257	0.179	0.152	0.315	0.331	0.241	0.136
Linear parallel trends	Yes	Yes	Yes	Yes	Yes	Yes	Yes	No	No	Yes	Yes
Non‐linear parallel trends	No	No	No	Yes	Yes	No	Yes	Yes	Yes	Yes	Yes
Observations	1827	1827	1827	1827	1827	1827	1827	1827	1827	1131	1131
Gaza	0.333	0.087	0.036	0.095	0.169***	0.079	0.256***	0.031	0.002	0.062	0.038
	(0.191)	(0.054)	(0.017)	(0.041)	(0.046)	(0.066)	(0.056)	(0.047)	(0.047)	(0.050)	(0.061)
R‐sq	0.128	0.099	0.113	0.137	0.164	0.110	0.143	0.175	0.164	0.445	0.229
Linear parallel trends	Yes	Yes	No	Yes	Yes	Yes	Yes	Yes	Yes	Yes	Yes
Non‐linear parallel trends	No	No	No	Yes	Yes	Yes	Yes	Yes	Yes	No	No
Observations	2110	2110	2110	2110	2110	2110	2110	2110	2110	1628	1628

*Note*: Constant, year of birth, month of birth, distance to health facility, squared distance to health facility, mothers' wealth quintile, years of education, 5‐year age‐group, number of children, Christian religion, household size, female head of household, age of head of household, rural residence, car ownership and fixed effects for closest health facility. DHS population weights applied to observations. Standard errors clustered at the DHS primary sampling unit level in parentheses.

*p < 0.10, **p < 0.05, ***p < 0.01, based on Bonferroni‐Holm *p*‐value.

### 5.4 Heterogenous effects by exposure

Table 5 shows that on average there were no significant improvements for populations within a reachable distance from a PBF facility. There were increases in HIV testing offered (16 pp, *p* = 0.008) and performed (24 pp, *p* < 0.001), in knowledge about HIV MTCT (21 pp, *p* = 0.018) and PMTCT (25 pp, *p* < 0.001), but only for those closest to a facility in the referral network of a PBF facility.

**TABLE 5:** Effects of performance based financing by exposure.

PBF exposure	Number of ANC visits	Four ANC visits	ANC with professional	Offered HIV test at ANC	Tested for HIV at ANC	Knowledge of HIV vertical transmission	Knowl. of HIV vert. transm. prevention	Institutional delivery	Institutional delivery with professional	Fully vaccinated 12 months	Fully vaccinated 9 months
PBF in closest HF	−0.024	0.047	−0.056	0.076	0.054	0.117	0.110	0.061	0.039	0.045	0.031
	(0.357)	(0.096)	(0.058)	(0.064)	(0.057)	(0.078)	(0.077)	(0.058)	(0.060)	(0.077)	(0.071)
R‐sq	0.341	0.243	0.398	0.400	0.421	0.233	0.253	0.445	0.451	0.410	0.290
Linear parallel trends	Yes	Yes	Yes	Yes	Yes	Yes	Yes	Yes	Yes	Yes	Yes
Non‐linear parallel trends	Yes	No	Yes	Yes	Yes	Yes	Yes	Yes	Yes	Yes	No
PBF in HF within 15 Km	0.199	0.094	−0.046	0.009	0.046	0.093	0.089	0.044	0.030	−0.020	0.002
	(0.266)	(0.078)	(0.041)	(0.056)	(0.054)	(0.061)	(0.061)	(0.045)	(0.047)	(0.074)	(0.066)
R‐sq	0.342	0.244	0.398	0.401	0.424	0.234	0.255	0.445	0.451	0.410	0.291
Linear parallel trends	Yes	Yes	Yes	Yes	Yes	Yes	Yes	Yes	Yes	Yes	No
Non‐linear parallel trends	Yes	No	Yes	Yes	Yes	Yes	Yes	Yes	Yes	Yes	Yes
PBF in referral facility	0.076	−0.010	−0.035	0.161***	0.241***	0.212**	0.246***	0.144	0.106	0.012	0.031
	(0.241)	(0.071)	(0.040)	(0.048)	(0.049)	(0.069)	(0.061)	(0.059)	(0.061)	(0.084)	(0.074)
R‐sq	0.342	0.242	0.398	0.401	0.424	0.235	0.256	0.446	0.451	0.410	0.290
Linear parallel trends	Yes	Yes	Yes	Yes	Yes	Yes	Yes	Yes	Yes	No	No
Non‐linear parallel trends	Yes	Yes	Yes	Yes	Yes	Yes	Yes	Yes	Yes	Yes	Yes
Observations	3937	3937	3937	3937	3937	3937	3937	3937	3937	2759	2759

*Note*: Constant, year of birth, month of birth, distance to health facility, squared distance to health facility, mothers' wealth quintile, years of education, 5‐year age‐group, number of children, Christian religion, household size, female head of household, age of head of household, rural residence, car ownership and fixed effects for closest health facility. DHS population weights applied to observations. Standard errors clustered at the DHS primary sampling unit level in parentheses.

*p < 0.10, **p < 0.05, ***p < 0.01, based on Bonferroni‐Holm *p*‐value.

Table 6 illustrates heterogeneity in the effects by wealth, education and provinces for different definitions of exposure to PBF. The improvements for women exposed via referral network were stronger for less wealthy in HIV testing offered (24 pp, *p* = 0.014), HIV testing performed (27 pp, *p* = 0.005), and knowledge about HIV MTCT (32 pp, *p* = 0.033) and PMTCT (30 pp, *p* = 0.005). For wealthier women there were smaller than average improvements, and only in HIV testing performed (24 pp, *p* = 0.013) and in knowledge about HIV PMTCT (27 pp, *p* < 0.001), when exposed via referral network. There were also smaller improvements in HIV testing (16 pp, *p* = 0.076) and in knowledge about PMTCT (18 pp, *p* = 0.065) for women within 15 Km from a PBF facility, and in knowledge about PMTCT (29 pp, *p* = 0.001) for women closest to a PBF facility. The improvements when exposed via referral network, were higher for less educated women in terms of HIV testing offered (18 pp, *p* = 0.009) and performed (27 pp, *p* = 0.006), and knowledge about HIV MTCT (37 pp, *p* = 0.0154) and PMTCT (43 pp, *p* < 0.001). There were also increases in knowledge about MTCT (24 pp, *p* = 0.081) for less educated women within 15 Km from a PBF facility. There were no effects for more educated women. In Nampula there were improvements in HIV testing performed (22 pp, *p* = 0.006) and in knowledge about HIV MTCT (25 pp, *p* = 0.079), for those exposed via referral network. In Gaza there were also improvements in HIV testing performed (23 pp, *p* = 0.019) and in knowledge about HIV PMTCT (31 pp, *p* = 0.026) for those exposed via referral network.

**TABLE 6:** Effects of performance based financing by exposure, wealth, education and province.

PBF exposure	Number of ANC visits	Four ANC visits	ANC with professional	Offered HIV test at ANC	Tested for HIV at ANC	Knowledge of HIV vertical transmission	Knowl. of HIV vert. transm. prevention	Institutional delivery	Institutional delivery with professional	Fully vaccinated 12 months	Fully vaccinated 9 months
Wealth											
Below median											
PBF in closest HF	−0.043	0.123	−0.133	0.078	0.073	0.126	0.087	0.076	0.065	0.014	0.020
	(0.500)	(0.127)	(0.094)	(0.100)	(0.093)	(0.101)	(0.087)	(0.094)	(0.096)	(0.095)	(0.096)
R‐sq	0.343	0.254	0.405	0.348	0.370	0.284	0.249	0.384	0.399	0.391	0.305
Linear parallel trends	Yes	Yes	Yes	Yes	Yes	Yes	Yes	Yes	Yes	Yes	Yes
Non‐linear parallel trends	Yes	Yes	Yes	Yes	Yes	Yes	No	No	Yes	Yes	No
PBF in HF within 15 Km	−0.166	0.101	−0.147	−0.006	−0.021	0.149	0.075	0.077	0.072	0.034	0.025
	(0.428)	(0.109)	(0.079)	(0.094)	(0.088)	(0.087)	(0.082)	(0.083)	(0.085)	(0.084)	(0.085)
R‐sq	0.343	0.255	0.405	0.349	0.371	0.285	0.250	0.384	0.399	0.392	0.306
Linear parallel trends	Yes	Yes	Yes	Yes	Yes	Yes	No	Yes	Yes	Yes	Yes
Non‐linear parallel trends	Yes	Yes	No	Yes	Yes	No	Yes	No	No	Yes	Yes
PBF in referral facility	−0.433	−0.123	−0.142	0.248**	0.273***	0.326**	0.299***	0.23	0.200	0.099	0.164
	(0.332)	(0.065)	(0.075)	(0.077)	(0.078)	(0.112)	(0.084)	(0.109)	(0.110)	(0.126)	(0.084)
R‐sq	0.343	0.254	0.405	0.351	0.373	0.288	0.252	0.386	0.400	0.392	0.307
Linear parallel trends	Yes	Yes	Yes	Yes	Yes	Yes	No	Yes	Yes	Yes	No
Non‐linear parallel trends	Yes	Yes	No	Yes	No	No	No	Yes	No	Yes	Yes
Observations	2020	2020	2020	2020	2020	2020	2020	2020	2020	1293	1293
Wealth											
Above median											
PBF in closest HF	0.143	−0.017	0.078	0.099	0.101	0.186	0.290***	0.041	−0.001	0.090	0.115
	(0.264)	(0.083)	(0.045)	(0.061)	(0.073)	(0.082)	(0.074)	(0.046)	(0.043)	(0.091)	(0.066)
R‐sq	0.312	0.287	0.351	0.368	0.380	0.273	0.322	0.396	0.371	0.549	0.396
Linear parallel trends	Yes	Yes	Yes	Yes	Yes	Yes	Yes	Yes	Yes	Yes	Yes
Non‐linear parallel trends	No	No	Yes	No	Yes	Yes	Yes	Yes	Yes	Yes	Yes
PBF in HF within 15 Km	0.546	0.124	0.073	0.040	0.162*	0.039	0.175**	0.059	0.036	−0.065	−0.017
	(0.238)	(0.103)	(0.034)	(0.063)	(0.060)	(0.074)	(0.063)	(0.041)	(0.045)	(0.089)	(0.078)
R‐sq	0.315	0.289	0.353	0.372	0.392	0.275	0.328	0.397	0.372	0.550	0.396
Linear parallel trends	Yes	Yes	Yes	Yes	Yes	Yes	Yes	Yes	Yes	Yes	Yes
Non‐linear parallel trends	Yes	Yes	Yes	Yes	Yes	Yes	Yes	Yes	Yes	Yes	Yes
PBF in referral facility	0.606	0.132	0.099	0.092	0.241**	0.102	0.272***	0.071	0.025	−0.019	−0.001
	(0.245)	(0.096)	(0.039)	(0.068)	(0.074)	(0.080)	(0.065)	(0.039)	(0.040)	(0.074)	(0.068)
R‐sq	0.314	0.288	0.353	0.368	0.385	0.272	0.323	0.396	0.371	0.549	0.395
Linear parallel trends	Yes	Yes	Yes	Yes	Yes	Yes	Yes	Yes	Yes	Yes	Yes
Non‐linear parallel trends	Yes	Yes	Yes	Yes	Yes	Yes	No	Yes	Yes	Yes	No
Observations	1917	1917	1917	1917	1917	1917	1917	1917	1917	1466	1466
Education											
0–4 years											
PBF in closest HF	0.026	0.083	−0.117	0.119	0.083	0.225	0.184	0.044	0.019	0.013	0.027
	(0.554)	(0.110)	(0.081)	(0.077)	(0.099)	(0.111)	(0.108)	(0.095)	(0.096)	(0.109)	(0.110)
R‐sq	0.379	0.283	0.431	0.405	0.415	0.276	0.271	0.413	0.426	0.406	0.318
Linear parallel trends	Yes	Yes	Yes	Yes	Yes	Yes	Yes	Yes	Yes	Yes	Yes
Non‐linear parallel trends	Yes	Yes	Yes	Yes	Yes	Yes	Yes	Yes	Yes	No	No
PBF in HF within 15 Km	−0.054	0.021	−0.107	0.018	0.052	0.242*	0.226	0.015	−0.006	−0.067	−0.023
	(0.449)	(0.098)	(0.063)	(0.073)	(0.087)	(0.090)	(0.096)	(0.079)	(0.080)	(0.103)	(0.101)
R‐sq	0.380	0.284	0.432	0.405	0.417	0.279	0.275	0.413	0.426	0.407	0.319
Linear parallel trends	Yes	Yes	Yes	Yes	Yes	Yes	Yes	Yes	Yes	Yes	Yes
Non‐linear parallel trends	Yes	Yes	Yes	Yes	Yes	Yes	Yes	Yes	Yes	Yes	Yes
PBF in referral facility	−0.120	−0.116	−0.056	0.182***	0.264***	0.367**	0.427***	0.148	0.104	0.038	0.025
	(0.306)	(0.072)	(0.064)	(0.055)	(0.065)	(0.117)	(0.072)	(0.097)	(0.099)	(0.128)	(0.103)
R‐sq	0.379	0.283	0.431	0.406	0.418	0.280	0.278	0.414	0.426	0.406	0.318
Linear parallel trends	Yes	Yes	Yes	Yes	Yes	Yes	No	Yes	Yes	Yes	No
Non‐linear parallel trends	No	No	No	Yes	Yes	No	Yes	Yes	Yes	Yes	Yes
Observations	2241	2241	2241	2241	2241	2241	2241	2241	2241	1486	1486
Education											
4 or more years											
PBF in closest HF	0.049	0.031	0.041	−0.045	−0.023	0.030	0.054	0.063	0.041	0.084	0.033
	(0.300)	(0.132)	(0.046)	(0.059)	(0.053)	(0.119)	(0.106)	(0.072)	(0.073)	(0.099)	(0.078)
R‐sq	0.353	0.334	0.330	0.443	0.430	0.369	0.392	0.537	0.514	0.611	0.476
Linear parallel trends	Yes	Yes	Yes	Yes	Yes	Yes	Yes	Yes	Yes	Yes	Yes
Non‐linear parallel trends	Yes	Yes	Yes	Yes	Yes	Yes	Yes	Yes	Yes	Yes	Yes
PBF in HF within 15 Km	0.493	0.162	0.023	−0.072	−0.025	−0.044	−0.068	0.038	0.035	0.024	0.033
	(0.265)	(0.109)	(0.037)	(0.059)	(0.071)	(0.089)	(0.069)	(0.058)	(0.060)	(0.105)	(0.082)
R‐sq	0.356	0.338	0.330	0.446	0.434	0.370	0.395	0.537	0.514	0.611	0.476
Linear parallel trends	Yes	Yes	Yes	Yes	Yes	Yes	Yes	Yes	Yes	Yes	Yes
Non‐linear parallel trends	Yes	Yes	Yes	Yes	Yes	Yes	Yes	Yes	Yes	Yes	Yes
PBF in referral facility	0.141	0.061	−0.025	0.037	0.163	0.101	0.095	0.054	0.012	−0.036	0.030
	(0.309)	(0.107)	(0.048)	(0.081)	(0.079)	(0.094)	(0.086)	(0.073)	(0.074)	(0.088)	(0.090)
R‐sq	0.353	0.334	0.329	0.443	0.432	0.370	0.393	0.537	0.514	0.611	0.476
Linear parallel trends	Yes	Yes	Yes	Yes	Yes	Yes	Yes	Yes	Yes	Yes	Yes
Non‐linear parallel trends	Yes	No	Yes	Yes	Yes	Yes	Yes	Yes	Yes	Yes	Yes
Observations	1696	1696	1696	1696	1696	1696	1696	1696	1696	1273	1273
Province											
Nampula											
PBF in closest HF	−0.251	0.032	−0.142	0.077	0.034	0.106	0.028	0.091	0.081	0.006	0.006
	(0.550)	(0.148)	(0.093)	(0.098)	(0.085)	(0.108)	(0.081)	(0.099)	(0.100)	(0.089)	(0.097)
R‐sq	0.319	0.229	0.392	0.336	0.349	0.255	0.216	0.432	0.456	0.344	0.265
Linear parallel trends	Yes	Yes	Yes	Yes	Yes	Yes	Yes	Yes	Yes	Yes	No
Non‐linear parallel trends	Yes	Yes	Yes	Yes	Yes	No	No	No	Yes	Yes	No
PBF in HF within 15 Km	0.077	0.142	−0.110	−0.070	−0.021	0.058	0.009	0.032	0.022	−0.088	−0.016
	(0.432)	(0.126)	(0.067)	(0.083)	(0.079)	(0.080)	(0.066)	(0.075)	(0.077)	(0.101)	(0.086)
R‐sq	0.319	0.231	0.392	0.339	0.351	0.255	0.217	0.432	0.456	0.345	0.267
Linear parallel trends	Yes	Yes	Yes	Yes	Yes	Yes	Yes	Yes	Yes	Yes	No
Non‐linear parallel trends	Yes	Yes	No	Yes	Yes	Yes	Yes	Yes	Yes	No	No
PBF in referral facility	0.015	−0.027	−0.091	0.159	0.222***	0.252*	0.205	0.171	0.139	−0.013	0.077
	(0.331)	(0.098)	(0.059)	(0.068)	(0.063)	(0.094)	(0.080)	(0.083)	(0.085)	(0.135)	(0.091)
R‐sq	0.318	0.229	0.392	0.337	0.351	0.258	0.219	0.434	0.456	0.344	0.266
Linear parallel trends	Yes	Yes	Yes	Yes	Yes	Yes	No	Yes	Yes	No	No
Non‐linear parallel trends	No	Yes	No	Yes	Yes	No	Yes	Yes	Yes	Yes	Yes
Observations	1827	1827	1827	1827	1827	1827	1827	1827	1827	1131	1131
Province											
Gaza											
PBF in closest HF	0.071	0.029	0.058*	0.063	0.088	0.130	0.263	0.054	0.006	0.018	0.002
	(0.194)	(0.058)	(0.024)	(0.058)	(0.074)	(0.090)	(0.110)	(0.060)	(0.057)	(0.077)	(0.078)
R‐sq	0.219	0.193	0.176	0.229	0.251	0.218	0.222	0.273	0.255	0.517	0.320
Linear parallel trends	Yes	Yes	Yes	Yes	Yes	Yes	Yes	Yes	Yes	Yes	Yes
Non‐linear parallel trends	No	No	No	Yes	Yes	Yes	Yes	Yes	Yes	No	No
PBF in HF within 15 Km	0.243	0.032	0.039	0.084	0.106	0.086	0.222	0.090	0.077	0.041	0.008
	(0.180)	(0.058)	(0.022)	(0.057)	(0.067)	(0.078)	(0.091)	(0.052)	(0.050)	(0.075)	(0.087)
R‐sq	0.220	0.193	0.177	0.232	0.259	0.220	0.232	0.274	0.256	0.518	0.320
Linear parallel trends	Yes	Yes	No	Yes	Yes	Yes	Yes	Yes	Yes	Yes	Yes
Non‐linear parallel trends	No	Yes	No	Yes	Yes	No	Yes	Yes	Yes	Yes	Yes
PBF in referral facility	0.201	0.002	0.063*	0.114	0.231**	0.107	0.307**	0.111*	0.070	0.081	0.041
	(0.184)	(0.054)	(0.028)	(0.061)	(0.073)	(0.077)	(0.101)	(0.053)	(0.051)	(0.072)	(0.084)
R‐sq	0.219	0.192	0.177	0.230	0.256	0.218	0.226	0.274	0.256	0.517	0.320
Linear parallel trends	Yes	Yes	No	Yes	Yes	Yes	Yes	yes	Yes	Yes	Yes
Non‐linear parallel trends	No	No	No	Yes	Yes	Yes	No	Yes	Yes	No	No
Observations	2110	2110	2110	2110	2110	2110	2110	2110	2110	1628	1628

*Note*: Constant, year of birth, month of birth, distance to health facility, squared distance to health facility, mothers' wealth quintile, years of education, 5‐year age‐group, number of children, Christian religion, household size, female head of household, age of head of household, rural residence, car ownership and fixed effects for closest health facility. DHS population weights applied to observations. Standard errors clustered at the DHS primary sampling unit level in parentheses.

*p < 0.10, **p < 0.05, ***p < 0.01, based on Bonferroni‐Holm *p*‐value.

### 5.5 Parallel trends test and robustness checks

Linear and non‐linear trends were parallel for most outcomes in the pre‐intervention period, with few exceptions for which we prefer not to rely on causal interpretation. Detailed test results are presented in Appendix (Figures A4 to A7 and Tables A2–A8). The results presented in Tables 3, 4, 5, 6 are reported with their corresponding unadjusted and adjusted *p*‐values in Table A10.

Results were robust to removing non‐exposed mothers within PBF provinces and within PBF districts, and to removing Maputo City from the control group. The sign and statistical significance of the coefficients remained unchanged when using non‐linear models for binary outcomes. None of the district characteristics in the pre‐intervention period was associated with PBF roll‐out, except for the percentage of economically active population and facility staffing levels (Table A9 in Appendix). When defining exposure by distance using a radium of 25 Km, the trends in outcomes pre‐intervention were often nonparallel, and no significant effects were found.

## 6 DISCUSSION

We evaluated the effects of a PBF scheme implemented in Gaza and Nampula provinces in Mozambique, on pre‐ and post‐natal maternal and child care, and on HIV testing and knowledge. The impact was limited. HIV testing during ANC increased, particularly for wealthier and more educated women, and in Gaza. Knowledge about MTCT and PMTCT also improved, particularly for less educated women, and in Nampula. Exploiting the program roll‐out by facility, and using alternative definitions of exposure, we investigated the role of health facilities' referral network and of district health administrations. HIV testing and knowledge did not improve for women in proximity of a PBF facility, but only for those whose closest facility is in the referral network of a PBF facility, or within the district more broadly. Antenatal care visits and institutional deliveries increased for mothers in the referral network of a PBF facility in Gaza. These results present a complex picture, with effects mainly in indicators related to the provision of HIV services and highly rewarded by incentives. Results suggest that PBF affected the whole local health system. There are signs that PBF incentives increased supply, and particularly for services functional to referring patients from within the whole district to PBF facilities. However, demand‐side constraints seemed to remain for less wealthy and less educated.

There is evidence of impact limited to increased HIV knowledge and testing, and of heterogenous effects within districts, which complements findings from Rajkotia et al. (2017), who merely assessed changes in the volume of incentivized services delivered by PBF facilities. The hypothesis that PBF facilities seek to stimulate demand for targeted and highly rewarded services through referral from lower to higher level facilities, is consistent with increased delivery of ART for pregnant women, ANC visits and PMTCT services in PBF facilities. We also found that this improvement was achieved through increased service use by women outside the immediate catchment area, but within the referral network, of PBF facilities, or within the district. This supports the hypothesis that outreach activities were carried out to stimulate demand and referral to increase service delivery in PBF facilities. Our results differ substantially for Nampula, where we did not find any evidence of increased ANC, nor institutional delivery, nor vaccinations. This difference suggests that increased service delivery may have been achieved at the intensive rather than extensive margin, or that effects may have weakened over the longer period covered in our study.

Although not directly targeted, the increase in HIV testing and knowledge may reflect attempts to identify target patients, and to boost demand and referral for highly rewarded HIV maternal and child care services. The increase in knowledge about HIV mother‐to‐child transmission, and about drugs that can prevent it, supports the hypothesis that efforts were made to stimulate demand and use of services in PBF facilities, rather than to increase knowledge per se. Improvements in knowledge and testing amongst the less educated are coherent with the hypothesis that outreach activities were undertaken amongst this group currently underusing services. However, the analysis by wealth reveals that there is an increase in service use, mostly testing, for wealthier women. This is consistent with evidence from other studies which highlight the role of demand‐side constraints in shaping health care seeking behavior, whereby even when knowledge has improved, only those who have the means increase their use of services (Binyaruka et al., 2018, 2020; Fichera et al., 2021; Van de Poel et al., 2016). Contextual demand and supply differences highlight the importance of structural demand‐side barriers to access and explain different results by province. Gaza, where effects are stronger, has a higher number of facilities, notably more resourced. The population is less sparse, wealthier, and better educated. Weaker effects in Nampula, suggest that incentives alone may not produce results, unless barriers to access, on both the demand and supply‐side, are addressed.

Exploiting the roll‐out by facility within the same district, we examined the effects on populations beyond the immediate catchment area of PBF facilities, and the possible mechanisms driving them. HIV testing and knowledge did not improve for mothers in proximity of a PBF facility, supporting the hypothesis that outreach activities were implemented through the referral network and under the coordination of the district administration. According to information provided by local policymakers, district administrations contribute to redistributing resources, including HIV tests, across facilities, to promote fairness and to support PBF facilities in achieving their targets. Tests may have been redistributed toward more peripheral areas for case finding and referral to PBF facilities for HIV treatment. Evidence of increased testing, particularly for wealthier women, supports the hypotheses that lower‐level facilities were engaged in stimulating demand, but changes were counter‐acted by demand‐side constraints for less wealthy women. Evidence of increased referral and outreach activities, with concentration of benefits in populations with higher socio‐economic status, was found in other settings (Fichera et al., 2021; Singh et al., 2021).

Data limitations constrained our analysis. Firstly, we had a limited set of outcomes, but we could assess population effects, not only on healthcare, but also on knowledge, using data of reliable quality. We were also limited in defining proximity based on a radius around the health facility, without accounting for access to main roads and transport. Although consistently with evidence from a qualitative study (Gergen et al., 2018), we could only hypothesize increased referral activities within the district, as we don't know from the data in which facility women sought care. Covariates were observed at the time of the interview, and not specifically at childbirth or conception. This may be a concern if PBF had an immediate effect on covariates, especially wealth and education, or if the mother moved between pregnancy and the interview. However, the DHS wealth index uses a wide range of assets which gives temporal‐robustness (Rutstein & Johnson, 2004). Educational status and the number of years at school are unlikely to change for mothers. We can not control for migration, like in other studies in the region, which use DHS data and making the same assumption (Bonfrer et al., 2014; Fichera et al., 2021; Sherry et al., 2017; Van de Poel et al., 2016). The DHS data do not report the facility where mothers sought care, so we relied on distance from facilities in the district to define exposure to PBF. Because the geo‐coordinate of the DHS cluster are randomly displaced, clusters could randomly incorrectly fall within a district, or an area around a facility or be incorrectly associated to the closest facility. Finally, we could not disentangle the effects over time using alternative study designs, nor control for every concurrent program implemented in the PBF districts. Although the major concurrent programmes were implemented on a national scale, the expansion of ART service provision to non PBF facilities may have been intensified in PBF districts, contributing to explain our results.

The impact of PBF remained limited to highly rewarded indicators, for which changes in the supply of services may have occurred, but with the potential impact reduced by demand‐side constraints impeding service use. Performance Based Financing can increase HIV testing during ANC, and the knowledge about prevention of HIV transmission from mother‐to‐child. These were not directly targeted by the program, but served to find patients and increase referral to PBF facilities for highly incentivized services. Increased efforts to stimulate demand may facilitate outreach to more disadvantaged populations within the whole district. However, increases in subsequent use of services, such as ANC and institutional deliveries, may be constrained by demand side or contextual characteristics. The impact may remain limited to wealthier populations, especially if patients are referred to more distant PBF facilities for incentivized services.

Further research using richer and more precise data is required to investigate the mechanisms which local health systems which could explain our results. However, we provide evidence that both health administrations and the way in which systems are organised shape the effects of PBF. We also provide evidence that structural contextual and supply factors, alongside demand‐side constraints, may limit the effectiveness of incentives to increase service use. Strengthening the capacity of local health systems and designing interventions which account for demand‐side constraints and for the organization of services, is key to achieving widespread access and improved service use, beyond targeted indicators and directly served populations.

## CONFLICT OF INTEREST

None.

## Supporting information

Supporting Information S1

## Data Availability

The data used are publicly available from The DHS program website or upon request from the National Institute of Health of Mozambique (INS).

## References

[hec4677-bib-0001] Angrist, J. D. , & Pischke, J.‐S. (2008). Mostly harmless econometrics: An empiricist's companion. Princeton University Press.

[hec4677-bib-0002] Anselmi, L. (2017). Relatório de mapeamento de recursos do sector da saúde no âmbito do apoio do mecanismo de financiamento global (Global Financing Facility ‐ GFF) para todas as mulheres e todas as crianças, Anexo II ao Caso de Investimento. Retrieved from https://www.globalfinancingfacility.org/sites/gff_new/files/documents/Mozambique‐IC‐Annexes‐PR.pdf

[hec4677-bib-0003] Anselmi, L. , Binyaruka, P. , & Borghi, J. (2017). Understanding causal pathways within health systems policy evaluation through mediation analysis: An application to payment for performance (P4P) in Tanzania. Implementation Science, 12(10), 10. 10.1186/s13012-016-0540-1 28148305 PMC5288944

[hec4677-bib-0004] Anselmi, L. , Lagarde, M. , & Hanson, K. (2015). Health service availability and health seeking behaviour in resource poor settings: Evidence from Mozambique. Health Economics Review, 5(1), 26. 10.1186/s13561-015-0062-6 26329425 PMC4556719

[hec4677-bib-0005] Anselmi, L. , Lagarde, M. , & Hanson, K. (2018). The efficiency of the local health systems: Investigating the roles of health administrations and health care providers. Health Economics, Policy and Law, 13(1), 10–32. 10.1017/s1744133117000068 28462745

[hec4677-bib-0006] Ashenfelter, O. (1978). Estimating the effect of training programs on earnings author. The Review of Economics and Statistics, 60(1), 47–57. 10.2307/1924332

[hec4677-bib-0007] Basinga, P. , Gertler, P. J. , Binagwaho, A. , Soucat, A. L. B. , Sturdy, J. , & Vermeersch, C. M. J. (2011). Effect on maternal and child health services in Rwanda of payment to primary health‐care providers for performance: An impact evaluation. The Lancet, 377(9775), 1421–1428. Elsevier Ltd. 10.1016/S0140-6736(11)60177-3 21515164

[hec4677-bib-0008] Benova, L. , Tunçalp, Ö. , Moran, A. C. , Maeve, O. , & Campbell, R. (2018). Not just a number: Examining coverage and content of antenatal care in low‐income and middle‐income countries. BMJ Global Health, 3(2), e000779. 10.1136/bmjgh-2018-000779 PMC5898334 29662698

[hec4677-bib-0009] Bertone, M. P. , Lurton, G. , & Mutombo, P. B. (2016). Investigating the remuneration of health workers in the DR Congo: Implications for the health workforce and the health system in a fragile setting. Health Policy and Planning, 31(9), 1143–1151. 10.1093/heapol/czv131 26758540

[hec4677-bib-0010] Bertone, M. P. , & Meessen, B. (2013). Studying the link between institutions and health system performance: A framework and an illustration with the analysis of two performance‐based financing schemes in Burundi. Health Policy and Planning, 28(8), 847–857. 10.1093/heapol/czs124 23221122

[hec4677-bib-0011] Binyaruka, P. , Lohmann, J. , & De Allegri, M. (2020). Evaluating performance‐based financing in low‐income and middle‐income countries: The need to look beyond average effect. BMJ Global Health, 5(8), e003136. 10.1136/bmjgh-2020-003136 PMC7418659 32784210

[hec4677-bib-0012] Binyaruka, P. , Patouillard, E. , Powell‐Jackson, T. , Greco, G. , Maestad, O. , & Borghi, J. (2015). Effect of paying for performance on utilisation, quality, and user costs of health services in Tanzania: A controlled before and after study. PLoS One, 10(8), e0135013. Public Library of Science. 26317510 10.1371/journal.pone.0135013 PMC4552688

[hec4677-bib-0013] Binyaruka, P. , Robberstad, B. , Torsvik, G. , & Borghi, J. (2018). Who benefits from increased service utilisation? Examining the distributional effects of payment for performance in Tanzania. International Journal for Equity in Health, 17(1), 14. 10.1186/s12939-018-0728-x 29378658 PMC5789643

[hec4677-bib-0014] Bonfrer, I. , Van de Poel, E. , & Van Doorslaer, E. (2014). The effects of performance incentives on the utilization and quality of maternal and child care in Burundi. Social Science and Medicine, 123, 96–104. 10.1016/j.socscimed.2014.11.004 25462610

[hec4677-bib-0015] Croft, T. N. , Marshall, A. M. J. , & Allen, C. K. , & DHS‐Team . (2018). Guide to DHS statistics. ICF.

[hec4677-bib-0016] Das, A. , Gopalan, S. S. , & Chandramohan, D. (2016). Effect of pay for performance to improve quality of maternal and child care in low‐ and middle‐income countries: A systematic review. BMC Public Health, 16(1), 321. 10.1186/s12889-016-2982-4 27074711 PMC4831162

[hec4677-bib-0017] De Allegri, M. , Bertone, M. P. , McMahon, S. , Mounpe Chare, I. , & Robyn, P. J. (2018). Unraveling PBF effects beyond impact evaluation: Results from a qualitative study in Cameroon. BMJ Global Health, 3(2), e000693. 10.1136/bmjgh-2017-000693 PMC5873544 29607103

[hec4677-bib-0018] Delgado, D. P. S (2013). Guião de Planificação e Gestão Para os Serviços Distritais de Saúde. Maputo.

[hec4677-bib-0019] DHS . (2020). GPS data collection. Retrieved from https://dhsprogram.com/What‐We‐Do/GPS‐Data‐Collection.cfm%0D. [Accessed on 9th May 2020].

[hec4677-bib-0020] Diaconu, K. , Falconer, J. , Verbel, A. , Fretheim, A. , & Witter, S. (2021). Paying for performance to improve the delivery of health interventions in low‐ and middle‐income countries. Cochrane Database of Systematic Reviews(5), CD007899. 33951190 10.1002/14651858.CD007899.pub3 PMC8099148

[hec4677-bib-0021] Eijkenaar, F. , Emmert, M. , Scheppach, M. , & Schöffski, O. (2013). Effects of pay for performance in health care: A systematic review of systematic reviews. Health Policy, 110(2–3), 115–130. Elsevier Ireland Ltd. 23380190 10.1016/j.healthpol.2013.01.008

[hec4677-bib-0022] Falisse, J.‐B. , Meessen, B. , Ndayishimiye, J. , & Bossuyt, M. (2012). Community participation and voice mechanisms under performance‐based financing schemes in Burundi. Tropical Medicine and International Health, 17(5), 674–682. John Wiley & Sons, Ltd. 22487362 10.1111/j.1365-3156.2012.02973.x

[hec4677-bib-0023] Falisse, J.‐B. , Ndayishimiye, J. , Kamenyero, V. , & Bossuyt, M. (2014). Performance‐based financing in the context of selective free health‐care: An evaluation of its effects on the use of primary health‐care services in Burundi using routine data. Health Policy and Planning, 30(10), 1251–1260. 10.1093/heapol/czu132 25533992

[hec4677-bib-0024] Fichera, E. , Anselmi, L. , Gwati, G. , Brown, G. , Kovacs, R. , & Borghi, J. (2021). Can results‐based financing improve health outcomes in resource poor settings? New evidence from Zimbabwe. Social Science and Medicine, 279, 113959. 10.1016/j.socscimed.2021.113959 33991792 PMC8210646

[hec4677-bib-0025] Francetic, I. , Tediosi, F. , & Kuwawenaruwa, A. (2020). A network analysis of patient referrals in two district health systems in Tanzania. Health Policy and Planning. 10.1093/heapol/czaa138 PMC7996649 33367559

[hec4677-bib-0026] Gage, A. , & Bauhoff, S. (2021). The effects of performance‐based financing on neonatal health outcomes in Burundi, Lesotho, Senegal, Zambia and Zimbabwe. Health Policy and Planning, 36(3), 332–340. 10.1093/heapol/czaa191 33491082 PMC8058947

[hec4677-bib-0027] Gergen, J. , Rajkotia, Y. , Lohmann, J. , & Ravishankar, N. (2018). Performance‐based financing kick‐starts motivational “feedback loop”: Findings from a process evaluation in Mozambique. Human Resources for Health, 16(55), 1–10. 30340497 10.1186/s12960-018-0320-x PMC6194661

[hec4677-bib-0028] Giacalone, M. , Agata, Z. , Cozzucoli, P. C. , & Alibrandi, A. (2018). Bonferroni‐holm and permutation tests to compare health data: Methodological and applicative issues. BMC Medical Research Methodology, 18(1), 81. 10.1186/s12874-018-0540-8 30029629 PMC6054729

[hec4677-bib-0029] Give, C. , Ndima, S. , Steege, R. , Ormel, H. , McCollum, R. , Theobald, S. , Taegtmeyer, M. , Kok, M. , & Sidat, M. (2019). Strengthening referral systems in community health programs: A qualitative study in two rural districts of Maputo province, Mozambique. BMC Health Services Research, 19(1), 263. 10.1186/s12913-019-4076-3 31035983 PMC6489304

[hec4677-bib-0030] Holm, S. (1979). A simple sequentially rejective multiple test procedure. Scandinavian Journal of Statistics, 6(2), 65–70. [Board of the Foundation of the Scandinavian Journal of Statistics, Wiley].

[hec4677-bib-0031] Instituto National de Saude (2018). Service Availability and Readiness Assessment (SARA) Mozambique. Maputo.

[hec4677-bib-0032] Jones, D. , Molitor, D. , & Reif, J. (2019). What do workplace wellness programs do? Evidence from the Illinois workplace wellness study. Quarterly Journal of Economics, 134(4), 1747–1791. 10.1093/qje/qjz023 31564754 PMC6756192

[hec4677-bib-0033] Kok, M. C. , Dieleman, M. , Taegtmeyer, M. , Broerse, J. E. W. , Kane, S. S. , Ormel, H. , Tijm, M. M. , & de Koning, K. A. M. (2015). Which intervention design factors influence performance of community health workers in low‐ and middle‐income countries? A systematic review. Health Policy and Planning, 30(9), 1207–1227. Oxford University Press. 10.1093/heapol/czu126 25500559 PMC4597042

[hec4677-bib-0034] Kovacs, R. J. , Powell‐Jackson, T. , Kristensen, S. R. , Singh, N. , & Borghi, J. (2020). How are pay‐for‐performance schemes in healthcare designed in low‐ and middle‐income countries? Typology and systematic literature review. BMC Health Services Research, 20(1), 291. 10.1186/s12913-020-05075-y 32264888 PMC7137308

[hec4677-bib-0035] Kuhnt, J. , & Vollmer, S. (2017). Antenatal care services and its implications for vital and health outcomes of children: Evidence from 193 surveys in 69 low‐income and middle‐income countries. BMJ Open, 7(11), 1–7. 10.1136/bmjopen-2017-017122 PMC5695442 29146636

[hec4677-bib-0036] Leonard, K. L. (2014). Active patients in rural African health care: Implications for research and policy. Health Policy and Planning, 29(1), 85–95. 10.1093/heapol/czs137 23307907

[hec4677-bib-0037] Martineau, T. , Raven, J. , Aikins, M. , Alonso‐Garbayo, A. , Baine, S. , Huss, R. , Maluka, S. , & Wyss, K. (2018). Strengthening health district management competencies in Ghana, Tanzania and Uganda: Lessons from using action research to improve health workforce performance. BMJ Global Health, 3(2), e000619, 10.1136/bmjgh-2017-000619 PMC5898347 29662692

[hec4677-bib-0038] Mendelson, A. , Kondo, K. , Damberg, C. , Low, A. , Motúapuaka, M. , Freeman, M. , O'Neil, M. , Relevo, R. , & Kansagara, D. (2017). The effects of pay‐for‐performance programs on health, health care use, and processes of care: A systematic review. Annals of Internal Medicine, 166(5), 341–353. 10.7326/m16-1881 28114600

[hec4677-bib-0039] Miller, G. , & Babiarz, K. S. (2013). Pay‐for‐Performance incentives in low‐ and middle‐income Country health programs. NBER Working Paper Series.

[hec4677-bib-0040] Ministério da Saúde (MISAU), Instituto Nacional de Estatística (INE), e ICF (2015). Inquérito de Indicadores de Imunização, Malária e HIV/SIDA em Moçambique 2015. Maputo: INS, INE.

[hec4677-bib-0041] MISAU . (2002). Diploma Ministerial 127/2002, de 31 de Julho (Regulamento que define a caracterização técnica e enunciado das funções do Serviço Nacional de Saúde), BR n. 14. Suplemento. Maputo.

[hec4677-bib-0042] MISAU . (2009). A needs assessment in maternal andNeonatal health inMozambique. Maputo.

[hec4677-bib-0043] MISAU . (2011). Moçambique Inquérito Demográfico e de Saúde 2011. Calverton.

[hec4677-bib-0044] MISAU . (2012). Revisao do sector saúde 2007–2012. Maputo.

[hec4677-bib-0045] MISAU . (2018). Inquérito de Indicadores de Imunização, Malária e HIV/SIDA em Moçambique ‐ IMASIDA, 2015. Maputo/Moçambique.

[hec4677-bib-0046] Nampula, D. P. S. (2018). Analise da situação de PBF em Nampula. Presentation prepared for Nampula Provincial Health Directorate.

[hec4677-bib-0047] Ngo, D. K. L. , Sherry, T. B. , & Bauhoff, S. (2016). Health system changes under pay‐for‐performance: The effects of Rwanda's national programme on facility inputs. Health Policy and Planning, 32(1), 11–20. 10.1093/heapol/czw091 27436339

[hec4677-bib-0048] Nimpagaritse, M. , Korachais, C. , Roberfroid, D. , Kolsteren, P. , Zine Eddine El Idrissi, M. D. , & Meessen, B. (2016). Measuring and understanding the effects of a performance based financing scheme applied to nutrition services in Burundi—A mixed method impact evaluation design. International Journal for Equity in Health, 15(1), 93. 10.1186/s12939-016-0382-0 27301741 PMC4908705

[hec4677-bib-0049] Peabody, J. , Shimkhada, R. , Quimbo, S. , Florentino, J. , Bacate, M. , McCulloch, C. E. , & Solon, O. (2011). Financial incentives and measurement improved physicians quality of care in the Philippines. Health Affairs, 30(4), 773–781. 10.1377/hlthaff.2009.0782 21471500

[hec4677-bib-0050] Rajkotia, Y. , Zang, O. , Nguimkeu, P. , Gergen, J. , Djurovic, I. , Vaz, P. , Mbofana, F. , Jobarteh, K. , Bassa, A. C. , & Bairro, F. (2017). The effect of a performance‐based financing program on HIV and maternal/child health services in Mozambique — An impact evaluation. Health Policy and Planning, 32(10), 1386–1396. 10.1093/heapol/czx106 29069378 PMC5886140

[hec4677-bib-0051] RBF Health . (2020). RBF health projects. Retrieved from https://www.rbfhealth.org/projects. [Accessed on 9th May 2020].

[hec4677-bib-0052] Renmans, D. , Holvoet, N. , Orach, C. G. , & Criel, B. (2016). Opening the “black box” of performance‐based financing in low‐ and lower middle‐income countries: A review of the literature. Health Policy and Planning, 31(9), 1297–1309. 10.1093/heapol/czw045 27126200

[hec4677-bib-0053] Rutstein, S. O. , & Johnson, K. (2004). The DHS wealth index. DHS Comparative Reports No. 6. Calverton.

[hec4677-bib-0054] Scott, A. , Liu, M. , & Yong, J. (2016). Financial incentives to encourage value‐based health care. Medical Care Research and Review, 75(1), 3–32. SAGE Publications Inc. 27815451 10.1177/1077558716676594

[hec4677-bib-0055] Scott, A. , Sivey, P. , Ait Ouakrim, D. , Willenberg, L. , Naccarella, L. , Furler, J. , & Young, D. (2011). The effect of financial incentives on the quality of health care provided by primary care physicians. Cochrane Database of Systematic Reviews (9). John Wiley and Sons, Ltd. 10.1002/14651858.CD008451.pub2 21901722

[hec4677-bib-0056] Sherry, T. , Bauhoff, S. , & Mohanan, M. (2017). Multitasking and heterogeneous treatment effects in pay‐for‐performance in health care: Evidence from Rwanda. American Journal of Health Economics, 3(2), 192–226. 10.1162/ajhe_a_00072

[hec4677-bib-0057] Singh, N. S. , Kovacs, R. J. , Cassidy, R. , Kristensen, S. R. , Borghi, J. , & Brown, G. W. (2021). A realist review to assess for whom, under what conditions and how pay for performance programmes work in low‐ and middle‐income countries. Social Science and Medicine, 270, 113624. 10.1016/j.socscimed.2020.113624 33373774

[hec4677-bib-0058] Soeters, R. , Peerenboom, P. B. , Mushagalusa, P. , & Kimanuka, C. (2011). Performance‐based financing experiment improved health care in the democratic republic of Congo. Health Affairs, 30(8), 1518–1527, 10.1377/hlthaff.2009.0019 21821568

[hec4677-bib-0059] Suthar, A. B. , Nagata, J. M. , Nsanzimana, S. , Bärnighausen, T. , Negussie, E. K. , & Doherty, M. C. (2017). Performance‐based financing for improving HIV/AIDS service delivery: A systematic review. BMC Health Services Research, 17(1), 6. 10.1186/s12913-016-1962-9 28052771 PMC5210258

[hec4677-bib-0060] Van de Poel, E. , Flores, G. , Ir, P. , & O'Donnell, O. (2016). Impact of performance‐based financing in a low‐resource setting: A decade of experience in Cambodia. Health Economics, 25(6), 688–705. 10.1002/hec.3219. John Wiley & Sons, Ltd 26224021

[hec4677-bib-0061] Wichman, C. J. (2017). Information provision and consumer behavior: A natural experiment in billing frequency. Journal of Public Economics, 152, 13–33. 10.1016/j.jpubeco.2017.05.004

[hec4677-bib-0062] Widmer, M. , Betran, A. P. , Merialdi, M. , Requejo, J. , & Karpf, T. (2011). The role of faith‐based organizations in maternal and newborn health care in Africa. International Journal of Gynecology & Obstetrics, 114(3), 218–222). World Health Organisation and Ireland Ltd. 21696730 10.1016/j.ijgo.2011.03.015

[hec4677-bib-0063] Witter, S. , Fretheim, A. , Kessey, F. & Lindahl, A. (2012). Paying for performance to improve the delivery of health interventions in low‐ and middle‐income countries. Cochrane Database of Systematic Reviews, 15(2). 10.1002/14651858.cd007899.pub2 22336833

[hec4677-bib-0064] Witter, S. , Toonen, J. , Meessen, B. , Kagubare, J. , Fritsche, G. , & Vaughan, K. (2013). Performance‐based financing as a health system reform: Mapping the key dimensions for monitoring and evaluation. BMC Health Services Research, 13(1), 367. 10.1186/1472-6963-13-367 24073625 PMC3849795

[hec4677-bib-0065] Zaresani, A. , & Scott, A. (2021). Is the evidence on the effectiveness of pay for performance schemes in healthcare changing? Evidence from a meta‐regression analysis. BMC Health Services Research, 21(1), 175. 10.1186/s12913-021-06118-8 33627112 PMC7905606

