# Supplementary material for: The impact of performance‐based financing within local health systems: Evidence from Mozambique
Source: Health Econ. 2023 Mar 27;32(7):1525–49. doi: 10.1002/hec.4677 (PMC10947248; doi:10.1002/hec.4677)
Supplement: Supplementary file 1 — Supporting Information S1 [file HEC-32-1525-s001.docx]

**Figure A1 - Health facilities in Mozambique, 2018**

**
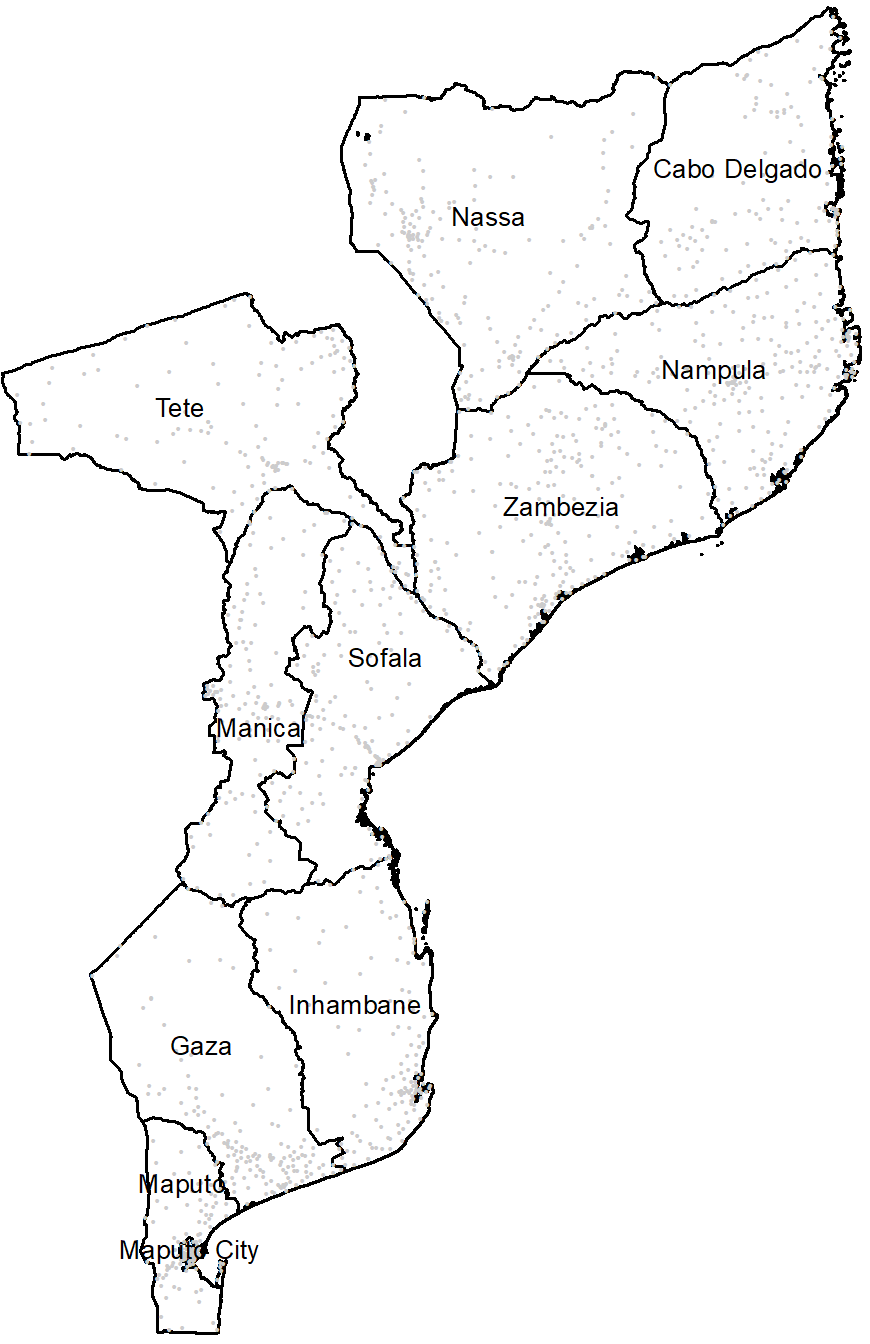
**

**Figure A2 – Gaza PBF roll-out by health facility**

**
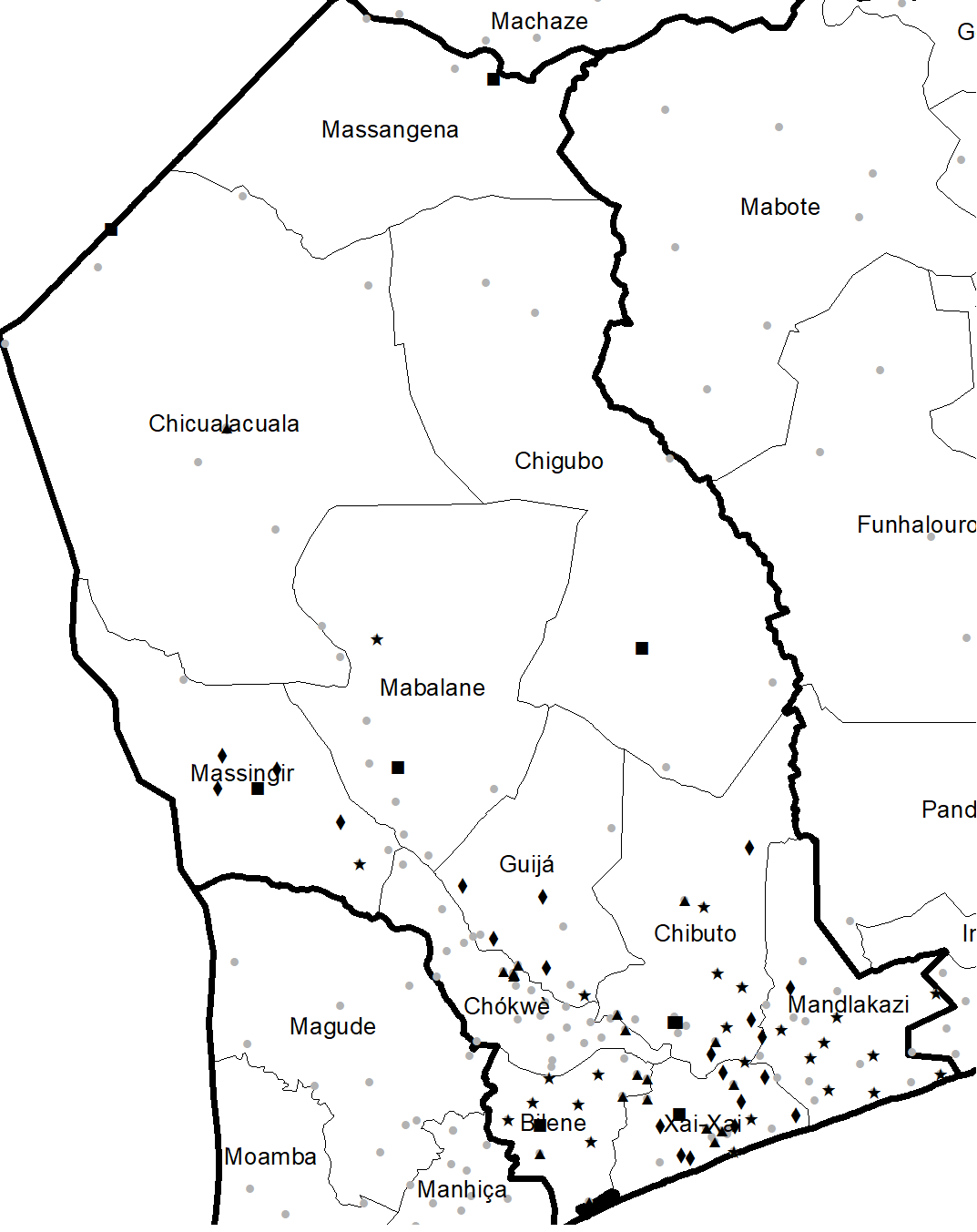
**

Phase 1: January 2011

Phase 2: March 2012

Phase 3: September 2013

Phase 4: September 2014

**Figure A3 – Nampula PBF roll-out by health facility**

**
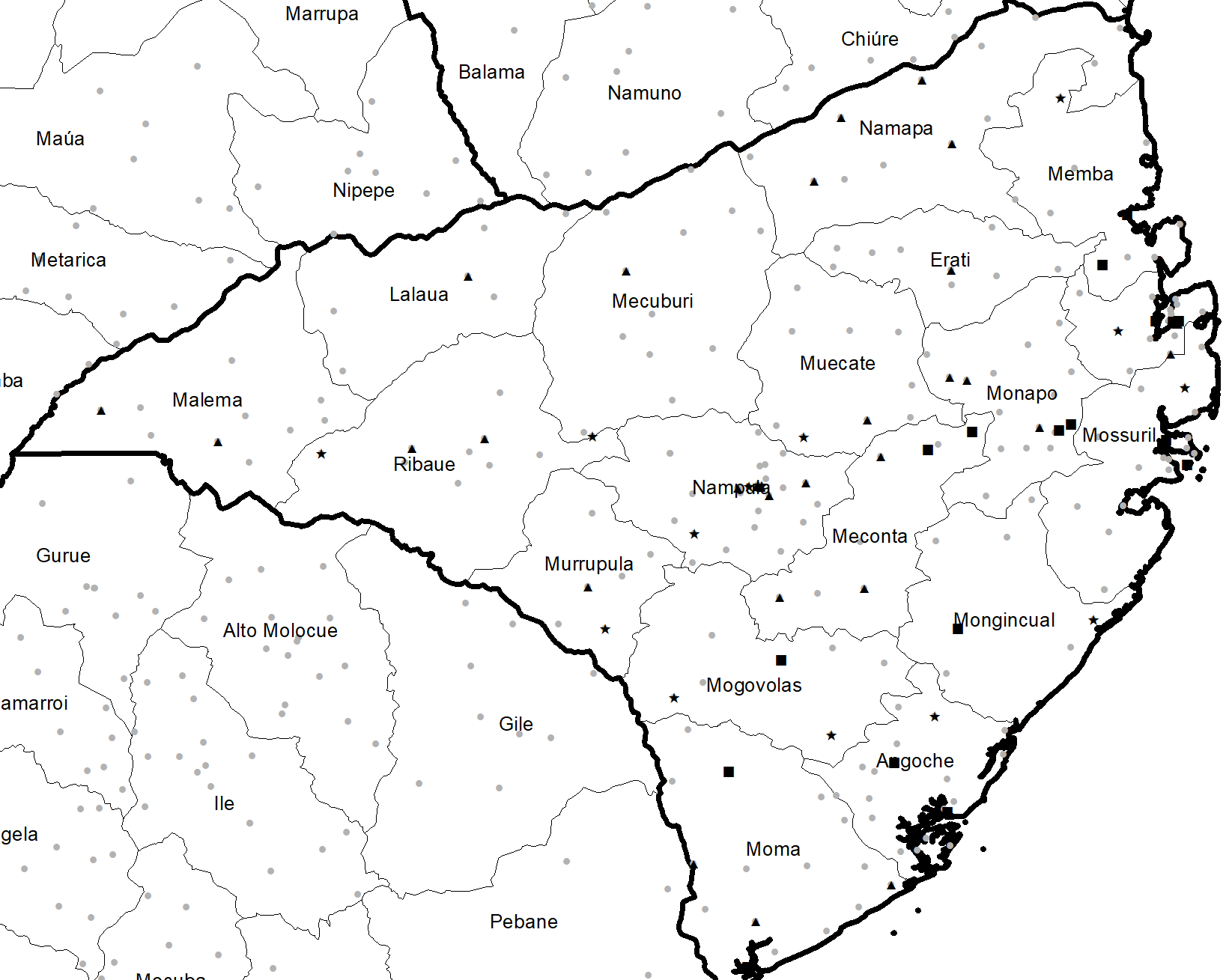
**

Phase 1: January 2011

Phase 2: March 2012

Phase 3: September 2013

Phase 4: September 2014

**Figure A4 Pre-intervention parallel trends test, PBF exposure by closest health facility, antenatal care and HIV knowledge**

**Figure A5 Pre-intervention parallel trends test, PBF exposure by health facility within 15 km, antenatal care and HIV knowledge**

**Figure A6 Pre-intervention parallel trends test, PBF exposure by referral health facility, antenatal care and HIV knowledge**

**Figure A7 Pre-intervention parallel trends test, PBF exposure by district, antenatal care and HIV knowledge**

**Table A.1 Descriptive statistics by exposure within Gaza and Nampula**

|  | **Gaza** | | | | **Nampula** | | | | | |
| --- | --- | --- | --- | --- | --- | --- | --- | --- | --- | --- |
|  | **Pre 2011** | | **Post 2011** | | **Pre 2011** | | | **Post 2011** | | |
| **Exposure by:** | **Never Exposed** | **Exposed** | **Never Exposed** | **Exposed** | **Never Exposed** | **Exposed** | **Never Exposed** | | **Exposed** |  |
| **District** |  |  |  |  |  |  |  | |  |  |
| Number of ANC visits |  | 4.09 | 4.40 | 4.55 | 3.69 | 3.75 | 3.76 | | 3.95 |  |
| Four ANC visits |  | 0.66 | 0.70 | 0.74 | 0.56 | 0.55 | 0.57 | | 0.59 |  |
| ANC with professional |  | 0.97 | 1.00 | 0.99 | 0.91 | 0.96 | 0.92 | | 0.95 |  |
| Offered HIV test at ANC |  | 0.75 | 0.90 | 0.88 | 0.70 | 0.67 | 0.72 | | 0.77 |  |
| Tested for HIV at ANC |  | 0.70 | 0.90 | 0.86 | 0.70 | 0.62 | 0.72 | | 0.75 |  |
| Knowledge of HIV vertical transmission |  | 0.63 | 0.90 | 0.63 | 0.71 | 0.63 | 0.62 | | 0.59 |  |
| Knowledge of HIV vertical tr. prevention |  | 0.71 | 0.90 | 0.81 | 0.77 | 0.68 | 0.69 | | 0.73 |  |
| Institutional delivery |  | 0.74 | 1.00 | 0.81 | 0.65 | 0.74 | 0.69 | | 0.72 |  |
| Institutional delivery with professional |  | 0.72 | 1.00 | 0.79 | 0.62 | 0.72 | 0.68 | | 0.71 |  |
| Fully vaccinated at 12 months |  | 0.67 | 0.43 | 0.41 | 0.59 | 0.64 | 0.28 | | 0.32 |  |
| Fully vaccinated at 9 months |  | 0.40 | 0.29 | 0.28 | 0.36 | 0.37 | 0.19 | | 0.21 |  |
| Observations |  | 453 | 10 | 344 | 4252 | 469 | 3070 | | 368 |  |
| **Closest Facility** |  |  |  |  |  |  |  | |  |  |
| Number of ANC visits | 4.12 | 4.06 | 4.72 | 4.40 | 3.67 | 3.81 | 3.75 | | 3.84 |  |
| Four ANC visits | 0.68 | 0.66 | 0.78 | 0.72 | 0.55 | 0.59 | 0.56 | | 0.58 |  |
| ANC with professional | 0.97 | 0.97 | 0.99 | 0.99 | 0.91 | 0.96 | 0.92 | | 0.96 |  |
| Offered HIV test at ANC | 0.76 | 0.75 | 0.84 | 0.91 | 0.69 | 0.66 | 0.71 | | 0.77 |  |
| Tested for HIV at ANC | 0.72 | 0.69 | 0.83 | 0.88 | 0.69 | 0.60 | 0.72 | | 0.75 |  |
| Knowledge of HIV vertical transmission | 0.63 | 0.63 | 0.62 | 0.64 | 0.70 | 0.66 | 0.61 | | 0.65 |  |
| Knowledge of HIV vertical tr. prevention | 0.73 | 0.69 | 0.79 | 0.83 | 0.76 | 0.65 | 0.69 | | 0.73 |  |
| Institutional delivery | 0.73 | 0.75 | 0.82 | 0.80 | 0.65 | 0.70 | 0.69 | | 0.71 |  |
| Institutional delivery with professional | 0.70 | 0.73 | 0.80 | 0.80 | 0.62 | 0.69 | 0.68 | | 0.70 |  |
| Fully vaccinated at 12 months | 0.72 | 0.64 | 0.37 | 0.44 | 0.59 | 0.61 | 0.28 | | 0.33 |  |
| Fully vaccinated at 9 months | 0.44 | 0.38 | 0.24 | 0.32 | 0.36 | 0.33 | 0.19 | | 0.22 |  |
| Observations | 186.00 | 267.00 | 156.00 | 198.00 | 4638 | 427 | 3431 | | 370 |  |
| **One facility within 15 Km** |  |  |  |  |  |  |  | |  |  |
| Number of ANC visits | 3.65 | 4.14 | 4.52 | 4.55 | 3.66 | 3.82 | 3.73 | | 3.94 |  |
| Four ANC visits | 0.52 | 0.68 | 0.77 | 0.73 | 0.55 | 0.59 | 0.56 | | 0.60 |  |
| ANC with professional | 0.96 | 0.97 | 1.00 | 0.99 | 0.91 | 0.96 | 0.92 | | 0.97 |  |
| Offered HIV test at ANC | 0.63 | 0.76 | 0.80 | 0.90 | 0.68 | 0.68 | 0.71 | | 0.79 |  |
| Tested for HIV at ANC | 0.54 | 0.72 | 0.80 | 0.87 | 0.69 | 0.63 | 0.71 | | 0.78 |  |
| Knowledge of HIV vertical transmission | 0.67 | 0.62 | 0.63 | 0.63 | 0.71 | 0.65 | 0.61 | | 0.65 |  |
| Knowledge of HIV vertical tr. prevention | 0.63 | 0.71 | 0.75 | 0.83 | 0.76 | 0.67 | 0.68 | | 0.75 |  |
| Institutional delivery | 0.41 | 0.78 | 0.89 | 0.79 | 0.64 | 0.74 | 0.69 | | 0.73 |  |
| Institutional delivery with professional | 0.41 | 0.75 | 0.87 | 0.78 | 0.61 | 0.73 | 0.67 | | 0.72 |  |
| Fully vaccinated at 12 months | 0.70 | 0.67 | 0.43 | 0.41 | 0.59 | 0.63 | 0.28 | | 0.31 |  |
| Fully vaccinated at 9 months | 0.45 | 0.40 | 0.26 | 0.29 | 0.36 | 0.36 | 0.19 | | 0.22 |  |
| Observations | 46 | 407 | 71 | 283 | 4431 | 634 | 3249 | | 552 |  |
| **Referral facility** |  |  |  |  |  |  |  | |  |  |
| Number of ANC visits |  | 4.09 | 4.40 | 4.55 | 3.69 | 3.69 | 3.77 | | 3.84 |  |
| Four ANC visits |  | 0.66 | 0.70 | 0.74 | 0.56 | 0.55 | 0.57 | | 0.56 |  |
| ANC with professional |  | 0.97 | 1.00 | 0.99 | 0.91 | 0.94 | 0.92 | | 0.94 |  |
| Offered HIV test at ANC |  | 0.75 | 0.90 | 0.88 | 0.70 | 0.64 | 0.72 | | 0.75 |  |
| Tested for HIV at ANC |  | 0.70 | 0.90 | 0.86 | 0.71 | 0.59 | 0.72 | | 0.74 |  |
| Knowledge of HIV vertical transmission |  | 0.63 | 0.90 | 0.63 | 0.71 | 0.65 | 0.62 | | 0.57 |  |
| Knowledge of HIV vertical tr. prevention |  | 0.71 | 0.90 | 0.81 | 0.78 | 0.66 | 0.69 | | 0.71 |  |
| Institutional delivery |  | 0.74 | 1.00 | 0.81 | 0.65 | 0.70 | 0.69 | | 0.69 |  |
| Institutional delivery with professional |  | 0.72 | 1.00 | 0.79 | 0.62 | 0.69 | 0.68 | | 0.68 |  |
| Fully vaccinated at 12 months |  | 0.67 | 0.43 | 0.41 | 0.59 | 0.63 | 0.28 | | 0.28 |  |
| Fully vaccinated at 9 months |  | 0.40 | 0.29 | 0.28 | 0.36 | 0.36 | 0.19 | | 0.19 |  |
| Observations |  | 453 | 10 | 344 | 4217 | 490 | 3057 | | 356 |  |

**Table A.2 Linear pre-intervention parallel trends test, Average**

| **PBF Exposure** | **Number of ANC visits** | **Four ANC visits** | **ANC with professional** | **Offered HIV test at ANC** | **Tested for HIV at ANC** | **Knowledge of HIV vertical transm.** | **Knowl. of HIV vert. transm. prevention** | **Institutional delivery** | **Institutional delivery with professional** | **Fully vaccinated at 12 months** | **Fully vaccinated at 9 months** |
| --- | --- | --- | --- | --- | --- | --- | --- | --- | --- | --- | --- |
| **PBF in closest HF** | -0.002 | 0.001 | -0.001 | 0.003 | 0.001 | 0.000 | -0.002 | 0.001 | 0.001 | 0.003 | 0.006 |
|  | (0.014) | (0.004) | (0.002) | (0.004) | (0.003) | (0.004) | (0.003) | (0.002) | (0.002) | (0.005) | (0.004) |
| R-sq | 0.336 | 0.248 | 0.420 | 0.421 | 0.449 | 0.194 | 0.271 | 0.487 | 0.492 | 0.351 | 0.292 |
|  |  |  |  |  |  |  |  |  |  |  |  |
| **PBF in HF within 15 Km** | -0.000 | 0.003 | -0.000 | 0.002 | 0.001 | -0.002 | -0.004 | 0.001 | 0.001 | 0.008 | 0.007* |
|  | (0.011) | (0.004) | (0.002) | (0.003) | (0.003) | (0.003) | (0.003) | (0.002) | (0.002) | (0.004) | (0.003) |
| R-sq | 0.336 | 0.249 | 0.420 | 0.421 | 0.449 | 0.194 | 0.272 | 0.487 | 0.493 | 0.354 | 0.293 |
|  |  |  |  |  |  |  |  |  |  |  |  |
| **PBF in referral facility** | 0.000 | 0.003 | 0.000 | 0.002 | 0.001 | -0.000 | -0.005 | 0.002 | 0.003 | 0.008* | 0.007** |
|  | (0.011) | (0.003) | (0.002) | (0.003) | (0.002) | (0.003) | (0.003) | (0.002) | (0.002) | (0.004) | (0.003) |
| R-sq | 0.336 | 0.249 | 0.420 | 0.421 | 0.449 | 0.194 | 0.273 | 0.488 | 0.493 | 0.355 | 0.294 |
|  |  |  |  |  |  |  |  |  |  |  |  |
| **PBF in District** | -0.000 | 0.000 | 0.000 | -0.000 | -0.000 | -0.001** | -0.000 | -0.001 | -0.001 | -0.000 | 0.001* |
|  | (0.001) | (0.000) | (0.000) | (0.000) | (0.000) | (0.000) | (0.001) | (0.000) | (0.000) | (0.000) | (0.000) |
| R-sq | 0.336 | 0.248 | 0.420 | 0.420 | 0.449 | 0.194 | 0.270 | 0.488 | 0.493 | 0.351 | 0.291 |
|  |  |  |  |  |  |  |  |  |  |  |  |
| Observations | 2217 | 2217 | 2217 | 2217 | 2217 | 2217 | 2217 | 2217 | 2217 | 1529 | 1529 |

Standard errors in parentheses; *** p<0.001, ** p<0.01, * p<0.5

**Table A.3 Linear pre-intervention parallel trends test, lower wealth quintiles**

| **Below Median Wealth PBF Exposure** | **Number of ANC visits** | **Four ANC visits** | **ANC with professional** | **Offered HIV test at ANC** | **Tested for HIV at ANC** | **Knowledge of HIV vertical transmission** | **Knowl. of HIV vert. transm. prevention** | **Institutional delivery** | **Institutional delivery with professional** | **Fully vaccinated at 12 months** | **Fully vaccinated at 9 months** |
| --- | --- | --- | --- | --- | --- | --- | --- | --- | --- | --- | --- |
| **PBF in closest HF** | -0.000 | 0.002 | -0.002 | 0.008 | 0.006 | -0.006 | -0.009 | 0.003 | 0.004 | 0.002 | 0.008 |
|  | (0.025) | (0.007) | (0.004) | (0.005) | (0.004) | (0.007) | (0.006) | (0.004) | (0.004) | (0.009) | (0.007) |
| R-sq | 0.355 | 0.278 | 0.443 | 0.351 | 0.374 | 0.221 | 0.266 | 0.434 | 0.450 | 0.386 | 0.307 |
|  |  |  |  |  |  |  |  |  |  |  |  |
| **PBF in HF within 15 Km** | -0.006 | 0.004 | -0.002 | 0.004 | 0.005 | -0.007 | -0.013** | 0.006 | 0.006 | 0.005 | 0.009 |
|  | (0.019) | (0.006) | (0.003) | (0.004) | (0.003) | (0.005) | (0.004) | (0.003) | (0.003) | (0.007) | (0.006) |
| R-sq | 0.355 | 0.279 | 0.443 | 0.350 | 0.374 | 0.223 | 0.271 | 0.436 | 0.452 | 0.386 | 0.309 |
|  |  |  |  |  |  |  |  |  |  |  |  |
| **PBF in referral of closest HF** | -0.005 | 0.004 | -0.002 | 0.003 | 0.004 | -0.002 | -0.011** | 0.005 | 0.006 | 0.008 | 0.010* |
|  | (0.016) | (0.005) | (0.003) | (0.003) | (0.003) | (0.004) | (0.004) | (0.003) | (0.003) | (0.006) | (0.004) |
| R-sq | 0.355 | 0.279 | 0.443 | 0.350 | 0.374 | 0.220 | 0.271 | 0.436 | 0.452 | 0.389 | 0.311 |
|  |  |  |  |  |  |  |  |  |  |  |  |
| **PBF in District** | -0.001 | -0.000 | -0.000 | 0.001 | 0.001* | -0.001* | -0.000 | -0.001 | -0.001 | 0.001 | 0.002* |
|  | (0.002) | (0.001) | (0.000) | (0.001) | (0.001) | (0.001) | (0.001) | (0.001) | (0.001) | (0.001) | (0.001) |
| R-sq | 0.355 | 0.278 | 0.443 | 0.350 | 0.374 | 0.221 | 0.262 | 0.434 | 0.450 | 0.386 | 0.307 |
|  |  |  |  |  |  |  |  |  |  |  |  |
| N | 1094 | 1094 | 1094 | 1094 | 1094 | 1094 | 1094 | 1094 | 1094 | 715 | 715 |

Standard errors in parentheses; *** p<0.001, ** p<0.01, * p<0.5

**Table A.4 Linear pre-intervention parallel trends test, higher wealth quintiles**

| **Above Median Wealth PBF Exposure** | **Number of ANC visits** | **Four ANC visits** | **ANC with professional** | **Offered HIV test at ANC** | **Tested for HIV at ANC** | **Knowledge of HIV vertical transm.** | **Knowl. of HIV vert. transm. prevention** | **Institutional delivery** | **Institutional delivery with professional** | **Fully vaccinated at 12 months** | **Fully vaccinated at 9 months** |
| --- | --- | --- | --- | --- | --- | --- | --- | --- | --- | --- | --- |
| **PBF in closest HF** | -0.015 | -0.004 | -0.001 | -0.002 | -0.002 | 0.007 | 0.007 | 0.000 | -0.001 | 0.006 | 0.002 |
|  | (0.014) | (0.005) | (0.002) | (0.004) | (0.004) | (0.004) | (0.003) | (0.003) | (0.003) | (0.004) | (0.004) |
| R-sq | 0.319 | 0.299 | 0.397 | 0.435 | 0.490 | 0.309 | 0.382 | 0.414 | 0.381 | 0.389 | 0.368 |
|  |  |  |  |  |  |  |  |  |  |  |  |
| **PBF in HF within 15 Km** | 0.001 | -0.000 | 0.001 | 0.001 | -0.000 | 0.005 | 0.004 | -0.002 | -0.002 | 0.008 | 0.001 |
|  | (0.013) | (0.005) | (0.002) | (0.004) | (0.003) | (0.004) | (0.003) | (0.003) | (0.003) | (0.004) | (0.004) |
| R-sq | 0.318 | 0.298 | 0.398 | 0.434 | 0.490 | 0.308 | 0.380 | 0.414 | 0.381 | 0.392 | 0.368 |
|  |  |  |  |  |  |  |  |  |  |  |  |
| **PBF in HF within 25 Km** | -0.003 | -0.001 | 0.001 | 0.002 | -0.001 | 0.005 | 0.004 | -0.001 | -0.001 | 0.008 | 0.001 |
|  | (0.013) | (0.004) | (0.002) | (0.004) | (0.003) | (0.004) | (0.003) | (0.003) | (0.003) | (0.004) | (0.004) |
| R-sq | 0.318 | 0.298 | 0.398 | 0.434 | 0.490 | 0.308 | 0.379 | 0.414 | 0.381 | 0.391 | 0.368 |
|  |  |  |  |  |  |  |  |  |  |  |  |
| **PBF in referral of closest HF** | 0.006 | 0.000 | 0.002 | 0.001 | -0.001 | 0.004 | 0.003 | 0.000 | 0.000 | 0.007 | 0.001 |
|  | (0.012) | (0.004) | (0.002) | (0.004) | (0.003) | (0.004) | (0.003) | (0.003) | (0.003) | (0.004) | (0.004) |
| R-sq | 0.318 | 0.298 | 0.399 | 0.434 | 0.490 | 0.307 | 0.378 | 0.414 | 0.381 | 0.390 | 0.368 |
|  |  |  |  |  |  |  |  |  |  |  |  |
| **PBF in District** | 0.006 | 0.000 | 0.002 | 0.001 | -0.001 | 0.004 | 0.003 | 0.000 | 0.000 | 0.007 | 0.001 |
|  | (0.012) | (0.004) | (0.002) | (0.004) | (0.003) | (0.004) | (0.003) | (0.003) | (0.003) | (0.004) | (0.004) |
| R-sq | 0.318 | 0.298 | 0.399 | 0.434 | 0.490 | 0.307 | 0.378 | 0.414 | 0.381 | 0.390 | 0.368 |
|  |  |  |  |  |  |  |  |  |  |  |  |
| N | 1123 | 1123 | 1123 | 1123 | 1123 | 1123 | 1123 | 1123 | 1123 | 814 | 814 |

Standard errors in parentheses; *** p<0.001, ** p<0.01, * p<0.5

**Table A.5 Linear pre-intervention parallel trends test, 0 to 4 years of education**

| **0-4 years of Education PBF Exposure** | **Number of ANC visits** | **Four ANC visits** | **ANC with professional** | **Offered HIV test at ANC** | **Tested for HIV at ANC** | **Knowledge of HIV vertical transmission** | **Knowl. of HIV vert. transm. prevention** | **Institutional delivery** | **Institutional delivery with professional** | **Fully vaccinated at 12 months** | **Fully vaccinated at 9 months** |
| --- | --- | --- | --- | --- | --- | --- | --- | --- | --- | --- | --- |
| **PBF in closest HF** | -0.002 | 0.003 | -0.001 | 0.001 | -0.001 | -0.004 | -0.003 | -0.001 | 0.000 | 0.002 | 0.003 |
|  | (0.018) | (0.005) | (0.004) | (0.003) | (0.004) | (0.005) | (0.005) | (0.004) | (0.004) | (0.007) | (0.006) |
| R-sq | 0.396 | 0.312 | 0.458 | 0.416 | 0.429 | 0.255 | 0.280 | 0.459 | 0.477 | 0.386 | 0.327 |
|  |  |  |  |  |  |  |  |  |  |  |  |
| **PBF in HF within 15 Km** | -0.002 | 0.004 | -0.000 | -0.001 | -0.001 | -0.006 | -0.007 | 0.000 | 0.001 | 0.008 | 0.008 |
|  | (0.015) | (0.005) | (0.003) | (0.003) | (0.003) | (0.004) | (0.004) | (0.003) | (0.003) | (0.006) | (0.005) |
| R-sq | 0.396 | 0.312 | 0.458 | 0.416 | 0.429 | 0.257 | 0.282 | 0.459 | 0.477 | 0.389 | 0.331 |
|  |  |  |  |  |  |  |  |  |  |  |  |
| **PBF in HF within 25 Km** | -0.000 | 0.000 | -0.000 | 0.000 | 0.000 | 0.001* | -0.001 | 0.001*** | 0.001*** | 0.001*** | -0.000 |
|  | (0.001) | (0.000) | (0.000) | (0.000) | (0.000) | (0.000) | (0.000) | (0.000) | (0.000) | (0.000) | (0.000) |
| R-sq | 0.396 | 0.311 | 0.458 | 0.416 | 0.429 | 0.255 | 0.280 | 0.460 | 0.478 | 0.388 | 0.327 |
|  |  |  |  |  |  |  |  |  |  |  |  |
| **PBF in referral of closest HF** | 0.004 | 0.005 | 0.001 | 0.000 | 0.000 | -0.002 | -0.007* | 0.002 | 0.003 | 0.008 | 0.010* |
|  | (0.013) | (0.004) | (0.003) | (0.003) | (0.003) | (0.004) | (0.004) | (0.003) | (0.003) | (0.005) | (0.004) |
| R-sq | 0.396 | 0.313 | 0.458 | 0.416 | 0.429 | 0.255 | 0.284 | 0.459 | 0.478 | 0.390 | 0.334 |
|  |  |  |  |  |  |  |  |  |  |  |  |
| **PBF in District** | 0.000 | 0.000 | 0.000 | -0.000 | -0.000 | -0.001*** | -0.000 | -0.001* | -0.001* | -0.001 | 0.001 |
|  | (0.001) | (0.000) | (0.000) | (0.000) | (0.000) | (0.000) | (0.001) | (0.000) | (0.000) | (0.000) | (0.000) |
| R-sq | 0.396 | 0.311 | 0.458 | 0.416 | 0.429 | 0.256 | 0.279 | 0.459 | 0.478 | 0.386 | 0.328 |
|  |  |  |  |  |  |  |  |  |  |  |  |
| N | 1328 | 1328 | 1328 | 1328 | 1328 | 1328 | 1328 | 1328 | 1328 | 895 | 895 |

Standard errors in parentheses; *** p<0.001, ** p<0.01, * p<0.5

**Table A.6 Linear pre-intervention parallel trends test, more than 4 years of education**

| **4 or more years of education PBF Exposure** | **Number of ANC visits** | **Four ANC visits** | **ANC**  **with**  **professional** | **Offered HIV test at ANC** | **Tested for HIV at ANC** | **Knowledge of HIV vertical**  **transmission** | **Knowl. of HIV vert. transm. prevention** | **Institutional**  **delivery** | **Institutional delivery with professional** | **Fully vaccinated at 12 months** | **Fully vaccinated at 9 months** |
| --- | --- | --- | --- | --- | --- | --- | --- | --- | --- | --- | --- |
| **PBF in closest HF** | -0.001 | 0.002 | -0.002 | 0.001 | 0.003 | 0.003 | 0.003 | 0.001 | -0.002 | 0.006 | 0.006 |
|  | (0.022) | (0.009) | (0.003) | (0.006) | (0.006) | (0.006) | (0.005) | (0.003) | (0.004) | (0.008) | (0.007) |
| R-sq | 0.378 | 0.398 | 0.388 | 0.522 | 0.519 | 0.376 | 0.451 | 0.615 | 0.581 | 0.547 | 0.497 |
|  |  |  |  |  |  |  |  |  |  |  |  |
| **PBF in HF within 15 Km** | 0.005 | 0.003 | -0.000 | 0.004 | 0.003 | 0.003 | 0.003 | 0.001 | 0.000 | 0.009 | 0.003 |
|  | (0.018) | (0.007) | (0.002) | (0.005) | (0.005) | (0.005) | (0.004) | (0.003) | (0.003) | (0.006) | (0.006) |
| R-sq | 0.378 | 0.399 | 0.388 | 0.523 | 0.520 | 0.376 | 0.451 | 0.615 | 0.580 | 0.549 | 0.496 |
|  |  |  |  |  |  |  |  |  |  |  |  |
| **PBF in HF within 25 Km** | -0.001 | 0.001 | -0.000 | 0.003 | 0.002 | 0.004 | 0.003 | 0.000 | -0.000 | 0.009 | 0.003 |
|  | (0.018) | (0.007) | (0.002) | (0.005) | (0.005) | (0.005) | (0.004) | (0.003) | (0.003) | (0.006) | (0.006) |
| R-sq | 0.378 | 0.398 | 0.388 | 0.523 | 0.519 | 0.376 | 0.451 | 0.615 | 0.580 | 0.549 | 0.496 |
|  |  |  |  |  |  |  |  |  |  |  |  |
| **PBF in referral of closest HF** | -0.000 | 0.000 | -0.000 | 0.004 | 0.003 | 0.003 | 0.002 | 0.002 | 0.001 | 0.009 | 0.005 |
|  | (0.017) | (0.007) | (0.002) | (0.005) | (0.005) | (0.005) | (0.004) | (0.003) | (0.004) | (0.006) | (0.006) |
| R-sq | 0.378 | 0.398 | 0.388 | 0.523 | 0.520 | 0.376 | 0.451 | 0.616 | 0.580 | 0.550 | 0.497 |
|  |  |  |  |  |  |  |  |  |  |  |  |
| **PBF in District** | -0.007 | -0.003 | -0.000 | 0.001 | -0.001 | 0.003 | 0.002 | 0.002 | 0.001 | 0.009 | 0.006 |
|  | (0.017) | (0.007) | (0.002) | (0.005) | (0.005) | (0.005) | (0.004) | (0.003) | (0.003) | (0.006) | (0.006) |
| R-sq | 0.378 | 0.399 | 0.388 | 0.522 | 0.519 | 0.376 | 0.451 | 0.616 | 0.580 | 0.550 | 0.497 |
|  |  |  |  |  |  |  |  |  |  |  |  |
| N | 889 | 889 | 889 | 889 | 889 | 889 | 889 | 889 | 889 | 634 | 634 |

Standard errors in parentheses; *** p<0.001, ** p<0.01, * p<0.5

**Table A.7 Linear pre-intervention parallel trends test, Nampula**

| **Nampula**  **PBF Exposure** | **Number of ANC visits** | **Four ANC visits** | **ANC with professional** | **Offered HIV test at ANC** | **Tested for HIV at ANC** | **Knowledge of HIV vertical transmission** | **Knowl. of HIV vert. transm. prevention** | **Institutio**  **nal**  **delivery** | **Institutional delivery with professional** | **Fully vaccinated at 12 months** | **Fully vaccinated at 9 months** |
| --- | --- | --- | --- | --- | --- | --- | --- | --- | --- | --- | --- |
| **PBF in closest HF** | -0.007 | -0.001 | -0.003 | 0.003 | -0.001 | -0.001 | -0.006 | 0.003 | 0.003 | 0.005 | 0.012* |
|  | (0.019) | (0.006) | (0.003) | (0.005) | (0.005) | (0.005) | (0.005) | (0.003) | (0.003) | (0.008) | (0.006) |
| R-sq | 0.343 | 0.268 | 0.421 | 0.359 | 0.358 | 0.190 | 0.243 | 0.495 | 0.525 | 0.340 | 0.305 |
|  |  |  |  |  |  |  |  |  |  |  |  |
| **PBF in HF within 15 Km** | -0.001 | 0.004 | -0.002 | 0.002 | -0.000 | -0.005 | -0.008 | 0.003 | 0.003 | 0.011 | 0.011* |
|  | (0.016) | (0.005) | (0.003) | (0.004) | (0.004) | (0.004) | (0.004) | (0.003) | (0.003) | (0.007) | (0.005) |
| R-sq | 0.343 | 0.269 | 0.421 | 0.358 | 0.358 | 0.192 | 0.245 | 0.495 | 0.526 | 0.344 | 0.305 |
|  |  |  |  |  |  |  |  |  |  |  |  |
| **PBF in HF within 25 Km** | -0.002 | -0.000 | -0.000 | 0.000 | -0.000 | 0.000 | -0.001 | 0.003*** | 0.003*** | 0.001 | 0.001 |
|  | (0.002) | (0.001) | (0.000) | (0.001) | (0.001) | (0.001) | (0.001) | (0.000) | (0.000) | (0.001) | (0.001) |
| R-sq | 0.343 | 0.268 | 0.420 | 0.358 | 0.359 | 0.190 | 0.242 | 0.498 | 0.529 | 0.339 | 0.297 |
|  |  |  |  |  |  |  |  |  |  |  |  |
| **PBF in referral of closest HF** | -0.002 | 0.004 | -0.001 | 0.003 | 0.001 | -0.002 | -0.008* | 0.003 | 0.003 | 0.012* | 0.011** |
|  | (0.015) | (0.005) | (0.003) | (0.004) | (0.003) | (0.004) | (0.004) | (0.003) | (0.003) | (0.006) | (0.004) |
| R-sq | 0.343 | 0.269 | 0.421 | 0.359 | 0.359 | 0.191 | 0.247 | 0.495 | 0.526 | 0.347 | 0.308 |
|  |  |  |  |  |  |  |  |  |  |  |  |
| **PBF in District** | 0.001 | 0.001 | -0.000 | 0.001 | 0.001 | -0.001 | -0.001 | -0.002** | -0.001* | 0.001 | 0.001 |
|  | (0.003) | (0.001) | (0.000) | (0.001) | (0.001) | (0.001) | (0.001) | (0.001) | (0.001) | (0.001) | (0.001) |
| R-sq | 0.343 | 0.269 | 0.421 | 0.358 | 0.359 | 0.191 | 0.241 | 0.496 | 0.526 | 0.340 | 0.298 |
|  |  |  |  |  |  |  |  |  |  |  |  |
| N | 975 | 975 | 975 | 975 | 975 | 975 | 975 | 975 | 975 | 617 | 617 |

Standard errors in parentheses; *** p<0.001, ** p<0.01, * p<0.5

**Table A.8 Linear pre-intervention parallel trends test, Gaza**

| **Gaza PBF Exposure** | **Number of ANC visits** | **Four ANC visits** | **ANC with professional** | **Offered HIV test at ANC** | **Tested for HIV at ANC** | **Knowledge of HIV vertical transmission** | **Knowl. of HIV vert. transm. prevention** | **Institutional delivery** | **Institutional delivery with professional** | **Fully vaccinated at 12 months** | **Fully vaccinated at 9 months** |
| --- | --- | --- | --- | --- | --- | --- | --- | --- | --- | --- | --- |
| **PBF in closest HF** | 0.009 | 0.005 | 0.004 | 0.002 | 0.003 | 0.004 | 0.006 | -0.004 | -0.004 | 0.004 | -0.001 |
|  | (0.015) | (0.004) | (0.002) | (0.002) | (0.003) | (0.004) | (0.004) | (0.003) | (0.003) | (0.004) | (0.003) |
| R-sq | 0.199 | 0.192 | 0.236 | 0.276 | 0.331 | 0.256 | 0.260 | 0.306 | 0.276 | 0.316 | 0.281 |
|  |  |  |  |  |  |  |  |  |  |  |  |
| **PBF in HF within 15 Km** | 0.001 | 0.000 | 0.003* | 0.001 | 0.001 | 0.002 | 0.000 | -0.004 | -0.003 | 0.004 | -0.003 |
|  | (0.011) | (0.003) | (0.002) | (0.002) | (0.002) | (0.003) | (0.004) | (0.003) | (0.003) | (0.003) | (0.003) |
| R-sq | 0.199 | 0.190 | 0.236 | 0.276 | 0.330 | 0.256 | 0.256 | 0.307 | 0.276 | 0.317 | 0.282 |
|  |  |  |  |  |  |  |  |  |  |  |  |
| **PBF in HF within 25 Km** | -0.002 | -0.000 | 0.003* | 0.001 | 0.000 | 0.003 | 0.000 | -0.004 | -0.003 | 0.004 | -0.003 |
|  | (0.011) | (0.003) | (0.002) | (0.002) | (0.002) | (0.003) | (0.003) | (0.003) | (0.003) | (0.003) | (0.003) |
| R-sq | 0.199 | 0.190 | 0.236 | 0.276 | 0.330 | 0.256 | 0.256 | 0.307 | 0.276 | 0.317 | 0.282 |
|  |  |  |  |  |  |  |  |  |  |  |  |
| **PBF in referral of closest HF** | 0.006 | 0.001 | 0.003* | 0.001 | 0.001 | 0.003 | -0.001 | -0.000 | 0.000 | 0.004 | -0.003 |
|  | (0.010) | (0.003) | (0.001) | (0.002) | (0.002) | (0.003) | (0.003) | (0.003) | (0.003) | (0.003) | (0.003) |
| R-sq | 0.199 | 0.191 | 0.236 | 0.276 | 0.330 | 0.257 | 0.256 | 0.305 | 0.275 | 0.317 | 0.282 |
|  |  |  |  |  |  |  |  |  |  |  |  |
| **PBF in District** | 0.006 | 0.001 | 0.003* | 0.001 | 0.001 | 0.003 | -0.001 | -0.000 | 0.000 | 0.004 | -0.003 |
|  | (0.010) | (0.003) | (0.001) | (0.002) | (0.002) | (0.003) | (0.003) | (0.003) | (0.003) | (0.003) | (0.003) |
| R-sq | 0.199 | 0.191 | 0.236 | 0.276 | 0.330 | 0.257 | 0.256 | 0.305 | 0.275 | 0.317 | 0.282 |
|  |  |  |  |  |  |  |  |  |  |  |  |
| N | 1242 | 1242 | 1242 | 1242 | 1242 | 1242 | 1242 | 1242 | 1242 | 912 | 912 |

Standard errors in parentheses; *** p<0.001, ** p<0.01, * p<0.5

**Table A9 – Association of district characteristics pre-2011 with PBF enrolment, logistic regression coefficients**

|  | **PBF versus No PBF with 2010 values** | **PBF versus No PBF with average 2008-2010 values** |
| --- | --- | --- |
| Weighted outpatient consultations per capita per year | 1.466 | 0.707 |
|  | (0.962) | (0.708) |
| Total number of health facilities per district | -0.036 | -0.050 |
|  | (0.279) | (0.224) |
| Average equipment to min stand | -0.016 | -0.003 |
|  | (0.021) | (0.026) |
| Average staff to min stand | -0.121** | -0.084** |
|  | (0.041) | (0.031) |
| Health facility housing availability (ration actual to norms) | 0.649 | 1.141 |
|  | (0.773) | (1.364) |
| District under 5-Year Mortality Rate | -0.002 | 0.003 |
|  | (0.020) | (0.019) |
| District recurrent expenditure state budget per health centre | 0.000 | 0.000 |
|  | (0.000) | (0.000) |
| District recurrent expenditure donor per health centre | -0.000* | -0.000* |
|  | (0.000) | (0.000) |
| District donor project expenditure per health centre | -0.000 | -0.000 |
|  | (0.000) | (0.000) |
| Economically active population (percentage) | 0.388** | 0.301** |
|  | (0.138) | (0.112) |
| Illiterate population (percentage) | -0.108 | -0.127 |
|  | (0.094) | (0.078) |
| District percentage children registered at birth | -0.045 | -0.034 |
|  | (0.025) | (0.020) |
| District percentage household with access to electricity | 0.112 | 0.032 |
|  | (0.272) | (0.195) |
| District % hhold with running water in/out house | 0.184 | 0.189 |
|  | (0.217) | (0.148) |
| District % hhold with no latrine or toilet | 0.100 | 0.093* |
|  | (0.052) | (0.043) |
| District % hhold with no items listed on asset index | 0.100 | 0.068 |
|  | (0.079) | (0.057) |
| Total population | 0.000 | 0.000 |
|  | (0.000) | (0.000) |
| Constant | -24.910* | -19.826* |
|  | (12.277) | (9.460) |
| Observations | 67 | 67 |
| Standard errors in parentheses; *** p<0.01, ** p<0.05, * p<0.1; The outcome variable is a binary variable, indicating if a district will be a PBF district. The variables in column (1) are 2010 values, except for under 5-year mortality rate which is recorded in 2007. The variables in column (2) are average over 2008, 2009 and 2010, except for under 5-year mortality rate which is recorded in 2007. | | |

**Table A10 –PBF effects (as in Tables 3 to 6) with unadjusted and adjusted p-values**

|  | | **Coefficient** | | **Standard Error** | | **p-Value** | | **Bonferroni-Holm p-value** | |
| --- | --- | --- | --- | --- | --- | --- | --- | --- | --- |
|  | |  | |  | |  | |  | |
| **Average effects of PBF** | | | | | | | | | |
| Number of ANC visits | | -0.1344 | | 0.1770 | | 0.4482 | | 1.0000 | |
| Four ANC visits | | -0.0073 | | 0.0494 | | 0.8818 | | 1.0000 | |
| ANC with professional | | -0.0417 | | 0.0311 | | 0.1815 | | 0.9074 | |
| Offered HIV test at ANC | | 0.0889 | | 0.0463 | | 0.0557 | | 0.4459 | |
| Tested for HIV at ANC | | 0.1341 | | 0.0464 | | 0.0041 | | **0.0372** | |
| Knowledge of HIV vertical transmission | | 0.1934 | | 0.0469 | | 0.0000 | | **0.0005** | |
| Knowl. of HIV vert. transm. prevention | | 0.3095 | | 0.0384 | | 0.0000 | | **0.0000** | |
| Institutional delivery | | -0.0270 | | 0.0451 | | 0.5499 | | 1.0000 | |
| Institutional delivery with professional | | -0.0580 | | 0.0451 | | 0.1996 | | 0.9074 | |
| Fully vaccinated 12 months | | -0.0591 | | 0.0396 | | 0.1369 | | 0.8212 | |
| Fully vaccinated 9 months | | -0.0602 | | 0.0362 | | 0.0967 | | 0.6771 | |
|  | |  | |  | |  | |  | |
| **Effects of PBF by wealth (below median)** | | | | | | | | | |
| Number of ANC visits | | -0.1979 | | 0.2492 | | 0.4278 | | 1.0000 | |
| Four ANC visits | | -0.0068 | | 0.0645 | | 0.9155 | | 1.0000 | |
| ANC with professional | | -0.0996 | | 0.0478 | | 0.0383 | | 0.3445 | |
| Offered HIV test at ANC | | 0.0825 | | 0.0692 | | 0.2343 | | 1.0000 | |
| Tested for HIV at ANC | | 0.0999 | | 0.0673 | | 0.1390 | | 1.0000 | |
| Knowledge of HIV vertical transmission | | 0.2791 | | 0.0623 | | 0.0000 | | **0.0001** | |
| Knowl. of HIV vert. transm. prevention | | 0.3799 | | 0.0524 | | 0.0000 | | **0.0000** | |
| Institutional delivery | | -0.0043 | | 0.0629 | | 0.9459 | | 1.0000 | |
| Institutional delivery with professional | | -0.0502 | | 0.0635 | | 0.4302 | | 1.0000 | |
| Fully vaccinated 12 months | | -0.0329 | | 0.0515 | | 0.5232 | | 1.0000 | |
| Fully vaccinated 9 months | | -0.0485 | | 0.0457 | | 0.2891 | | 1.0000 | |
|  | |  | |  | |  | |  | |
| **Effects of PBF by wealth (above median)** | | | | | | | | | |
| Number of ANC visits | | 0.0565 | | 0.1944 | | 0.7715 | | 1.0000 | |
| Four ANC visits | | 0.0176 | | 0.0692 | | 0.7997 | | 1.0000 | |
| ANC with professional | | 0.0528 | | 0.0244 | | 0.0311 | | 0.2487 | |
| Offered HIV test at ANC | | 0.1176 | | 0.0484 | | 0.0158 | | 0.1426 | |
| Tested for HIV at ANC | | 0.1925 | | 0.0489 | | 0.0001 | | **0.0010** | |
| Knowledge of HIV vertical transmission | | 0.0796 | | 0.0601 | | 0.1860 | | 1.0000 | |
| Knowl. of HIV vert. transm. prevention | | 0.2152 | | 0.0495 | | 0.0000 | | **0.0002** | |
| Institutional delivery | | -0.0512 | | 0.0425 | | 0.2295 | | 1.0000 | |
| Institutional delivery with professional | | -0.0721 | | 0.0432 | | 0.0963 | | 0.6743 | |
| Fully vaccinated 12 months | | -0.0493 | | 0.0543 | | 0.3643 | | 1.0000 | |
| Fully vaccinated 9 months | | -0.0252 | | 0.0571 | | 0.6595 | | 1.0000 | |
|  | |  | |  | |  | |  | |
|  | | **Coefficient** | | **Standard Error** | | **p-Value** | | **Bonferroni-Holm p-value** | |
| **Effects of PBF by education (below median)** | | | | | | | | | |
| Number of ANC visits | | -0.1345 | | 0.2302 | | 0.5594 | | 1.0000 | |
| Four ANC visits | | -0.0412 | | 0.0596 | | 0.4897 | | 1.0000 | |
| ANC with professional | | -0.0280 | | 0.0433 | | 0.5178 | | 1.0000 | |
| Offered HIV test at ANC | | 0.0978 | | 0.0657 | | 0.1373 | | 0.9613 | |
| Tested for HIV at ANC | | 0.1260 | | 0.0663 | | 0.0583 | | 0.4667 | |
| Knowledge of HIV vertical transmission | | 0.2916 | | 0.0650 | | 0.0000 | | **0.0001** | |
| Knowl. of HIV vert. transm. prevention | | 0.4277 | | 0.0532 | | 0.0000 | | **0.0000** | |
| Institutional delivery | | 0.0126 | | 0.0607 | | 0.8354 | | 1.0000 | |
| Institutional delivery with professional | | -0.0214 | | 0.0596 | | 0.7203 | | 1.0000 | |
| Fully vaccinated 12 months | | -0.0520 | | 0.0539 | | 0.3346 | | 1.0000 | |
| Fully vaccinated 9 months | | -0.0927 | | 0.0459 | | 0.0442 | | 0.3981 | |
|  | |  | |  | |  | |  | |
| **Effects of PBF by education (above median)** | | | | | | | | | |
| Number of ANC visits | | -0.0597 | | 0.2001 | | 0.7656 | | 1.0000 | |
| Four ANC visits | | 0.0122 | | 0.0707 | | 0.8636 | | 1.0000 | |
| ANC with professional | | 0.0024 | | 0.0257 | | 0.9243 | | 1.0000 | |
| Offered HIV test at ANC | | 0.0768 | | 0.0525 | | 0.1449 | | 1.0000 | |
| Tested for HIV at ANC | | 0.1349 | | 0.0511 | | 0.0087 | | **0.0959** | |
| Knowledge of HIV vertical transmission | | 0.0239 | | 0.0545 | | 0.6621 | | 1.0000 | |
| Knowl. of HIV vert. transm. prevention | | 0.1121 | | 0.0513 | | 0.0295 | | 0.2953 | |
| Institutional delivery | | -0.0733 | | 0.0511 | | 0.1525 | | 1.0000 | |
| Institutional delivery with professional | | -0.0942 | | 0.0507 | | 0.0638 | | 0.5740 | |
| Fully vaccinated 12 months | | -0.0936 | | 0.0567 | | 0.0994 | | 0.7953 | |
| Fully vaccinated 9 months | | 0.0114 | | 0.0549 | | 0.8358 | | 1.0000 | |
|  | |  | |  | |  | |  | |
| \|  \| **Coefficient** \| **Standard Error** \| **p-Value** \| **Bonferroni-Holm p-value** \| \| --- \| --- \| --- \| --- \| --- \| | | | | | | | | | |
| **Effects of PBF by province (Nampula)** | | | | | | | | | |
| Number of ANC visits | | -0.2516 | | 0.2394 | | 0.2948 | | 1.0000 | |
| Four ANC visits | | -0.0430 | | 0.0675 | | 0.5254 | | 1.0000 | |
| ANC with professional | | -0.0815 | | 0.0480 | | 0.0914 | | 0.8226 | |
| Offered HIV test at ANC | | 0.0578 | | 0.0680 | | 0.3964 | | 1.0000 | |
| Tested for HIV at ANC | | 0.0882 | | 0.0668 | | 0.1884 | | 0.9706 | |
| Knowledge of HIV vertical transmission | | 0.3000 | | 0.0572 | | 0.0000 | | **0.0000** | |
| Knowl. of HIV vert. transm. prevention | | 0.3667 | | 0.0499 | | 0.0000 | | **0.0000** | |
| Institutional delivery | | -0.0487 | | 0.0583 | | 0.4047 | | 1.0000 | |
| Institutional delivery with professional | | -0.0818 | | 0.0582 | | 0.1618 | | 0.9706 | |
| Fully vaccinated 12 months | | -0.1028 | | 0.0607 | | 0.0924 | | 0.8226 | |
| Fully vaccinated 9 months | | -0.0830 | | 0.0495 | | 0.0957 | | 0.8226 | |
|  | |  | |  | |  | |  | |
| **Effects of PBF by province (Gaza)** | | | | | | | | | |
| Number of ANC visits | | 0.3331 | | 0.1913 | | 0.0829 | | 0.5803 | |
| Four ANC visits | | 0.0870 | | 0.0537 | | 0.1068 | | 0.6408 | |
| ANC with professional | | 0.0359 | | 0.0167 | | 0.0332 | | 0.2657 | |
| Offered HIV test at ANC | | 0.0948 | | 0.0408 | | 0.0211 | | 0.1900 | |
| Tested for HIV at ANC | | 0.1686 | | 0.0458 | | 0.0003 | | **0.0029** | |
| Knowledge of HIV vertical transmission | | 0.0789 | | 0.0655 | | 0.2299 | | 1.0000 | |
| Knowl. of HIV vert. transm. prevention | | 0.2563 | | 0.0559 | | 0.0000 | | **0.0001** | |
| Institutional delivery | | 0.0313 | | 0.0471 | | 0.5065 | | 1.0000 | |
| Institutional delivery with professional | | 0.0022 | | 0.0471 | | 0.9622 | | 1.0000 | |
| Fully vaccinated 12 months | | 0.0619 | | 0.0499 | | 0.2164 | | 1.0000 | |
| Fully vaccinated 9 months | | 0.0377 | | 0.0611 | | 0.5371 | | 1.0000 | |
|  | |  | |  | |  | |  | |
|  | |  | |  | |  | |  | |
|  | | **Coefficient** | | **Standard Error** | | **p-Value** | | **Bonferroni-Holm p-value** | |
| **Effects of PBF by Exposure** | | | | | | | | | |
| **Health Facility** | | | | | | | | | |
| Number of ANC visits | | -0.0236 | | 0.3573 | | 0.9473 | | 1.0000 | |
| Four ANC visits | | 0.0466 | | 0.0957 | | 0.6267 | | 1.0000 | |
| ANC with professional | | -0.0563 | | 0.0576 | | 0.3291 | | 1.0000 | |
| Offered HIV test at ANC | | 0.0755 | | 0.0637 | | 0.2362 | | 1.0000 | |
| Tested for HIV at ANC | | 0.0540 | | 0.0574 | | 0.3482 | | 1.0000 | |
| Knowledge of HIV vertical transmission | | 0.1173 | | 0.0784 | | 0.1357 | | 1.0000 | |
| Knowl. of HIV vert. transm. prevention | | 0.1104 | | 0.0765 | | 0.1500 | | 1.0000 | |
| Institutional delivery | | 0.0607 | | 0.0580 | | 0.2963 | | 1.0000 | |
| Institutional delivery with professional | | 0.0386 | | 0.0602 | | 0.5216 | | 1.0000 | |
| Fully vaccinated 12 months | | 0.0446 | | 0.0771 | | 0.5631 | | 1.0000 | |
| Fully vaccinated 9 months | | 0.0314 | | 0.0709 | | 0.6576 | | 1.0000 | |
| **Health Facility within 15 km** | | | | | | | | | |
| Number of ANC visits | | 0.1989 | | 0.2659 | | 0.4549 | | 1.0000 | |
| Four ANC visits | | 0.0944 | | 0.0783 | | 0.2284 | | 1.0000 | |
| ANC with professional | | -0.0463 | | 0.0410 | | 0.2593 | | 1.0000 | |
| Offered HIV test at ANC | | 0.0089 | | 0.0560 | | 0.8739 | | 1.0000 | |
| Tested for HIV at ANC | | 0.0456 | | 0.0544 | | 0.4020 | | 1.0000 | |
| Knowledge of HIV vertical transmission | | 0.0931 | | 0.0609 | | 0.1269 | | 1.0000 | |
| Knowl. of HIV vert. transm. prevention | | 0.0886 | | 0.0609 | | 0.1462 | | 1.0000 | |
| Institutional delivery | | 0.0440 | | 0.0451 | | 0.3297 | | 1.0000 | |
| Institutional delivery with professional | | 0.0304 | | 0.0472 | | 0.5202 | | 1.0000 | |
| Fully vaccinated 12 months | | -0.0204 | | 0.0737 | | 0.7827 | | 1.0000 | |
| Fully vaccinated 9 months | | 0.0024 | | 0.0662 | | 0.9706 | | 1.0000 | |
| **Referral health facility** | | | | | | | | | |
| Number of ANC visits | | 0.0758 | | 0.2414 | | 0.7538 | | 1.0000 | |
| Four ANC visits | | -0.0102 | | 0.0708 | | 0.8853 | | 1.0000 | |
| ANC with professional | | -0.0354 | | 0.0402 | | 0.3782 | | 1.0000 | |
| Offered HIV test at ANC | | 0.1611 | | 0.0485 | | 0.0010 | | **0.0088** | |
| Tested for HIV at ANC | | 0.2407 | | 0.0486 | | 0.0000 | | **0.0000** | |
| Knowledge of HIV vertical transmission | | 0.2121 | | 0.0688 | | 0.0022 | | **0.0178** | |
| Knowl. of HIV vert. transm. prevention | | 0.2465 | | 0.0614 | | 0.0001 | | **0.0007** | |
| Institutional delivery | | 0.1436 | | 0.0586 | | 0.0147 | | **0.1030** | |
| Institutional delivery with professional | | 0.1062 | | 0.0610 | | 0.0825 | | 0.4951 | |
| Fully vaccinated 12 months | | 0.0125 | | 0.0842 | | 0.8821 | | 1.0000 | |
| Fully vaccinated 9 months | | 0.0312 | | 0.0744 | | 0.6752 | | 1.0000 | |
|  | |  | |  | |  | |  | |
| \|  \| **Coefficient** \| **Standard Error** \| **p-Value** \| **Bonferroni-Holm p-value** \| \| --- \| --- \| --- \| --- \| --- \| | | | | | | | | | |
| **Health Facility** | | | | | | | | | |
| Number of ANC visits | | -0.0426 | | 0.5005 | | 0.9323 | | 1.0000 | |
| Four ANC visits | | 0.1229 | | 0.1270 | | 0.3341 | | 1.0000 | |
| ANC with professional | | -0.1325 | | 0.0940 | | 0.1597 | | 1.0000 | |
| Offered HIV test at ANC | | 0.0782 | | 0.0998 | | 0.4339 | | 1.0000 | |
| Tested for HIV at ANC | | 0.0732 | | 0.0934 | | 0.4339 | | 1.0000 | |
| Knowledge of HIV vertical transmission | | 0.1256 | | 0.1013 | | 0.2163 | | 1.0000 | |
| Knowl. of HIV vert. transm. prevention | | 0.0871 | | 0.0872 | | 0.3187 | | 1.0000 | |
| Institutional delivery | | 0.0756 | | 0.0941 | | 0.4221 | | 1.0000 | |
| Institutional delivery with professional | | 0.0653 | | 0.0959 | | 0.4964 | | 1.0000 | |
| Fully vaccinated 12 months | | 0.0136 | | 0.0954 | | 0.8864 | | 1.0000 | |
| Fully vaccinated 9 months | | 0.0197 | | 0.0961 | | 0.8377 | | 1.0000 | |
| **Health Facility within 15 km** | | | | | | | | | |
| Number of ANC visits | | -0.1658 | | 0.4282 | | 0.6990 | | 1.0000 | |
| Four ANC visits | | 0.1008 | | 0.1086 | | 0.3541 | | 1.0000 | |
| ANC with professional | | -0.1468 | | 0.0789 | | 0.0641 | | 0.7053 | |
| Offered HIV test at ANC | | -0.0061 | | 0.0943 | | 0.9481 | | 1.0000 | |
| Tested for HIV at ANC | | -0.0207 | | 0.0880 | | 0.8138 | | 1.0000 | |
| Knowledge of HIV vertical transmission | | 0.1488 | | 0.0871 | | 0.0887 | | 0.8870 | |
| Knowl. of HIV vert. transm. prevention | | 0.0748 | | 0.0816 | | 0.3599 | | 1.0000 | |
| Institutional delivery | | 0.0774 | | 0.0832 | | 0.3530 | | 1.0000 | |
| Institutional delivery with professional | | 0.0720 | | 0.0848 | | 0.3969 | | 1.0000 | |
| Fully vaccinated 12 months | | 0.0337 | | 0.0837 | | 0.6874 | | 1.0000 | |
| Fully vaccinated 9 months | | 0.0249 | | 0.0848 | | 0.7692 | | 1.0000 | |
| **Referral health faciliy** | | | | | | | | | |
| Number of ANC visits | | -0.4332 | | 0.3321 | | 0.1933 | | 0.3865 | |
| Four ANC visits | | -0.1232 | | 0.0647 | | 0.0580 | | 0.3160 | |
| ANC with professional | | -0.1416 | | 0.0750 | | 0.0603 | | 0.3160 | |
| Offered HIV test at ANC | | 0.2478 | | 0.0772 | | 0.0015 | | **0.0135** | |
| Tested for HIV at ANC | | 0.2725 | | 0.0776 | | 0.0005 | | **0.0052** | |
| Knowledge of HIV vertical transmission | | 0.3260 | | 0.1125 | | 0.0041 | | **0.0325** | |
| Knowl. of HIV vert. transm. prevention | | 0.2992 | | 0.0842 | | 0.0005 | | **0.0050** | |
| Institutional delivery | | 0.2303 | | 0.1094 | | 0.0363 | | 0.2539 | |
| Institutional delivery with professional | | 0.1998 | | 0.1104 | | 0.0714 | | 0.3160 | |
| Fully vaccinated 12 months | | 0.0994 | | 0.1263 | | 0.4322 | | 0.4322 | |
| Fully vaccinated 9 months | | 0.1642 | | 0.0843 | | 0.0527 | | 0.3160 | |
|  | |  | |  | |  | |  | |
| \|  \| **Coefficient** \| **Standard Error** \| **p-Value** \| **Bonferroni-Holm p-value** \| \| --- \| --- \| --- \| --- \| --- \| | | | | | | | | | |
| **Health Facility** | | | | | | | | | |
| Number of ANC visits | | 0.1425 | | 0.2637 | | 0.5892 | | 1.0000 | |
| Four ANC visits | | -0.0168 | | 0.0825 | | 0.8384 | | 1.0000 | |
| ANC with professional | | 0.0779 | | 0.0446 | | 0.0817 | | 0.7353 | |
| Offered HIV test at ANC | | 0.0990 | | 0.0610 | | 0.1057 | | 0.7399 | |
| Tested for HIV at ANC | | 0.1009 | | 0.0734 | | 0.1702 | | 1.0000 | |
| Knowledge of HIV vertical transmission | | 0.1862 | | 0.0823 | | 0.0244 | | 0.2441 | |
| Knowl. of HIV vert. transm. prevention | | 0.2902 | | 0.0743 | | 0.0001 | | **0.0013** | |
| Institutional delivery | | 0.0407 | | 0.0455 | | 0.3718 | | 1.0000 | |
| Institutional delivery with professional | | -0.0008 | | 0.0433 | | 0.9852 | | 1.0000 | |
| Fully vaccinated 12 months | | 0.0898 | | 0.0910 | | 0.3245 | | 1.0000 | |
| Fully vaccinated 9 months | | 0.1149 | | 0.0661 | | 0.0830 | | 0.7353 | |
| **Health Facility within 15 km** | | | | | | | | | |
| Number of ANC visits | | 0.5460 | | 0.2381 | | 0.0226 | | 0.2030 | |
| Four ANC visits | | 0.1236 | | 0.1028 | | 0.2305 | | 1.0000 | |
| ANC with professional | | 0.0725 | | 0.0335 | | 0.0313 | | 0.2504 | |
| Offered HIV test at ANC | | 0.0400 | | 0.0631 | | 0.5268 | | 1.0000 | |
| Tested for HIV at ANC | | 0.1624 | | 0.0604 | | 0.0076 | | **0.0757** | |
| Knowledge of HIV vertical transmission | | 0.0393 | | 0.0738 | | 0.5943 | | 1.0000 | |
| Knowl. of HIV vert. transm. prevention | | 0.1748 | | 0.0630 | | 0.0059 | | **0.0645** | |
| Institutional delivery | | 0.0592 | | 0.0413 | | 0.1523 | | 1.0000 | |
| Institutional delivery with professional | | 0.0357 | | 0.0447 | | 0.4253 | | 1.0000 | |
| Fully vaccinated 12 months | | -0.0652 | | 0.0893 | | 0.4659 | | 1.0000 | |
| Fully vaccinated 9 months | | -0.0174 | | 0.0778 | | 0.8234 | | 1.0000 | |
| **Referral health facility** | | | | | | | | | |
| Number of ANC visits | | 0.6063 | | 0.2451 | | 0.0140 | | 0.1116 | |
| Four ANC visits | | 0.1316 | | 0.0964 | | 0.1730 | | 1.0000 | |
| ANC with professional | | 0.0988 | | 0.0390 | | 0.0118 | | 0.1060 | |
| Offered HIV test at ANC | | 0.0916 | | 0.0685 | | 0.1824 | | 1.0000 | |
| Tested for HIV at ANC | | 0.2412 | | 0.0744 | | 0.0013 | | **0.0132** | |
| Knowledge of HIV vertical transmission | | 0.1018 | | 0.0797 | | 0.2025 | | 1.0000 | |
| Knowl. of HIV vert. transm. prevention | | 0.2717 | | 0.0655 | | 0.0000 | | **0.0005** | |
| Institutional delivery | | 0.0708 | | 0.0394 | | 0.0738 | | 0.5168 | |
| Institutional delivery with professional | | 0.0246 | | 0.0402 | | 0.5414 | | 1.0000 | |
| Fully vaccinated 12 months | | -0.0193 | | 0.0742 | | 0.7949 | | 1.0000 | |
| Fully vaccinated 9 months | | -0.0012 | | 0.0682 | | 0.9854 | | 1.0000 | |
|  | |  | |  | |  | |  | |
| \|  \| **Coefficient** \| **Standard Error** \| **p-Value** \| **Bonferroni-Holm p-value** \| \| --- \| --- \| --- \| --- \| --- \| | | | | | | | | | |
| **Health Facility** | | | | | | | | | |
| Number of ANC visits | | 0.0260 | | 0.5538 | | 0.9625 | | 1.0000 | |
| Four ANC visits | | 0.0834 | | 0.1098 | | 0.4483 | | 1.0000 | |
| ANC with professional | | -0.1169 | | 0.0808 | | 0.1488 | | 1.0000 | |
| Offered HIV test at ANC | | 0.1188 | | 0.0767 | | 0.1222 | | 1.0000 | |
| Tested for HIV at ANC | | 0.0826 | | 0.0990 | | 0.4050 | | 1.0000 | |
| Knowledge of HIV vertical transmission | | 0.2252 | | 0.1113 | | 0.0439 | | 0.4830 | |
| Knowl. of HIV vert. transm. prevention | | 0.1843 | | 0.1083 | | 0.0898 | | 0.8976 | |
| Institutional delivery | | 0.0437 | | 0.0952 | | 0.6463 | | 1.0000 | |
| Institutional delivery with professional | | 0.0193 | | 0.0965 | | 0.8419 | | 1.0000 | |
| Fully vaccinated 12 months | | 0.0131 | | 0.1093 | | 0.9047 | | 1.0000 | |
| Fully vaccinated 9 months | | 0.0274 | | 0.1101 | | 0.8040 | | 1.0000 | |
| **Health Facility within 15 km** | | | | | | | | | |
| Number of ANC visits | | -0.0539 | | 0.4494 | | 0.9046 | | 1.0000 | |
| Four ANC visits | | 0.0210 | | 0.0980 | | 0.8305 | | 1.0000 | |
| ANC with professional | | -0.1068 | | 0.0632 | | 0.0920 | | 0.8281 | |
| Offered HIV test at ANC | | 0.0182 | | 0.0727 | | 0.8030 | | 1.0000 | |
| Tested for HIV at ANC | | 0.0521 | | 0.0871 | | 0.5501 | | 1.0000 | |
| Knowledge of HIV vertical transmission | | 0.2420 | | 0.0897 | | 0.0073 | | **0.0807** | |
| Knowl. of HIV vert. transm. prevention | | 0.2257 | | 0.0964 | | 0.0198 | | 0.1975 | |
| Institutional delivery | | 0.0152 | | 0.0791 | | 0.8475 | | 1.0000 | |
| Institutional delivery with professional | | -0.0061 | | 0.0801 | | 0.9396 | | 1.0000 | |
| Fully vaccinated 12 months | | -0.0671 | | 0.1028 | | 0.5147 | | 1.0000 | |
| Fully vaccinated 9 months | | -0.0232 | | 0.1013 | | 0.8193 | | 1.0000 | |
| **Referral health facility** | | | | | | | | | |
| Number of ANC visits | | -0.1200 | | 0.3065 | | 0.6957 | | 1.0000 | |
| Four ANC visits | | -0.1159 | | 0.0721 | | 0.1089 | | 0.7623 | |
| ANC with professional | | -0.0555 | | 0.0635 | | 0.3824 | | 1.0000 | |
| Offered HIV test at ANC | | 0.1822 | | 0.0550 | | 0.0010 | | **0.0092** | |
| Tested for HIV at ANC | | 0.2639 | | 0.0650 | | 0.0001 | | **0.0006** | |
| Knowledge of HIV vertical transmission | | 0.3674 | | 0.1175 | | 0.0019 | | **0.0154** | |
| Knowl. of HIV vert. transm. prevention | | 0.4266 | | 0.0720 | | 0.0000 | | **0.0000** | |
| Institutional delivery | | 0.1481 | | 0.0971 | | 0.1282 | | 0.7692 | |
| Institutional delivery with professional | | 0.1040 | | 0.0986 | | 0.2924 | | 1.0000 | |
| Fully vaccinated 12 months | | 0.0382 | | 0.1279 | | 0.7656 | | 1.0000 | |
| Fully vaccinated 9 months | | 0.0252 | | 0.1028 | | 0.8064 | | 1.0000 | |
|  |  | |  | |  | |  | |  |
|  |  | |  | |  | |  | |  |
|  | **Coefficient** | | **Standard Error** | | **p-Value** | | **Bonferroni-Holm p-value** | |  |
| **Effects of PBF by Exposure and Education (above median)** | | | | | | | | | |
| **Health Facility** | | | | | | | | | |
| Number of ANC visits | | 0.0491 | | 0.3003 | | 0.8703 | | 1.0000 | |
| Four ANC visits | | 0.0309 | | 0.1323 | | 0.8154 | | 1.0000 | |
| ANC with professional | | 0.0414 | | 0.0463 | | 0.3724 | | 1.0000 | |
| Offered HIV test at ANC | | -0.0454 | | 0.0591 | | 0.4430 | | 1.0000 | |
| Tested for HIV at ANC | | -0.0229 | | 0.0534 | | 0.6677 | | 1.0000 | |
| Knowledge of HIV vertical transmission | | 0.0296 | | 0.1192 | | 0.8037 | | 1.0000 | |
| Knowl. of HIV vert. transm. prevention | | 0.0542 | | 0.1063 | | 0.6107 | | 1.0000 | |
| Institutional delivery | | 0.0628 | | 0.0720 | | 0.3841 | | 1.0000 | |
| Institutional delivery with professional | | 0.0408 | | 0.0732 | | 0.5780 | | 1.0000 | |
| Fully vaccinated 12 months | | 0.0843 | | 0.0989 | | 0.3944 | | 1.0000 | |
| Fully vaccinated 9 months | | 0.0333 | | 0.0776 | | 0.6683 | | 1.0000 | |
| **Health Facility within 15 km** | | | | | | | | | |
| Number of ANC visits | | 0.4933 | | 0.2653 | | 0.0638 | | 0.7017 | |
| Four ANC visits | | 0.1619 | | 0.1095 | | 0.1402 | | 1.0000 | |
| ANC with professional | | 0.0227 | | 0.0375 | | 0.5449 | | 1.0000 | |
| Offered HIV test at ANC | | -0.0716 | | 0.0590 | | 0.2262 | | 1.0000 | |
| Tested for HIV at ANC | | -0.0246 | | 0.0707 | | 0.7286 | | 1.0000 | |
| Knowledge of HIV vertical transmission | | -0.0439 | | 0.0887 | | 0.6209 | | 1.0000 | |
| Knowl. of HIV vert. transm. prevention | | -0.0681 | | 0.0690 | | 0.3244 | | 1.0000 | |
| Institutional delivery | | 0.0380 | | 0.0577 | | 0.5111 | | 1.0000 | |
| Institutional delivery with professional | | 0.0349 | | 0.0597 | | 0.5594 | | 1.0000 | |
| Fully vaccinated 12 months | | 0.0239 | | 0.1053 | | 0.8203 | | 1.0000 | |
| Fully vaccinated 9 months | | 0.0333 | | 0.0821 | | 0.6857 | | 1.0000 | |
| **Referral health facility** | | | | | | | | | |
| Number of ANC visits | | 0.1411 | | 0.3090 | | 0.6483 | | 1.0000 | |
| Four ANC visits | | 0.0611 | | 0.1073 | | 0.5695 | | 1.0000 | |
| ANC with professional | | -0.0254 | | 0.0483 | | 0.5997 | | 1.0000 | |
| Offered HIV test at ANC | | 0.0369 | | 0.0809 | | 0.6487 | | 1.0000 | |
| Tested for HIV at ANC | | 0.1634 | | 0.0792 | | 0.0398 | | 0.4373 | |
| Knowledge of HIV vertical transmission | | 0.1014 | | 0.0942 | | 0.2826 | | 1.0000 | |
| Knowl. of HIV vert. transm. prevention | | 0.0951 | | 0.0863 | | 0.2713 | | 1.0000 | |
| Institutional delivery | | 0.0536 | | 0.0725 | | 0.4606 | | 1.0000 | |
| Institutional delivery with professional | | 0.0122 | | 0.0740 | | 0.8689 | | 1.0000 | |
| Fully vaccinated 12 months | | -0.0360 | | 0.0881 | | 0.6826 | | 1.0000 | |
| Fully vaccinated 9 months | | 0.0298 | | 0.0895 | | 0.7393 | | 1.0000 | |
|  | |  | |  | |  | |  | |
|  | | **Coefficient** | | **Standard Error** | | **p-Value** | | **Bonferroni-Holm p-value** | |
| **Effects of PBF by Exposure and Province (Nampula)** | | | | | | | | | |
| **Health Facility** | | | | | | | | | |
| Number of ANC visits | | -0.2513 | | 0.5503 | | 0.6485 | | 1.0000 | |
| Four ANC visits | | 0.0315 | | 0.1475 | | 0.8312 | | 1.0000 | |
| ANC with professional | | -0.1423 | | 0.0926 | | 0.1263 | | 1.0000 | |
| Offered HIV test at ANC | | 0.0768 | | 0.0983 | | 0.4357 | | 1.0000 | |
| Tested for HIV at ANC | | 0.0339 | | 0.0848 | | 0.6902 | | 1.0000 | |
| Knowledge of HIV vertical transmission | | 0.1060 | | 0.1077 | | 0.3265 | | 1.0000 | |
| Knowl. of HIV vert. transm. prevention | | 0.0284 | | 0.0809 | | 0.7262 | | 1.0000 | |
| Institutional delivery | | 0.0907 | | 0.0990 | | 0.3607 | | 1.0000 | |
| Institutional delivery with professional | | 0.0814 | | 0.1000 | | 0.4170 | | 1.0000 | |
| Fully vaccinated 12 months | | 0.0060 | | 0.0890 | | 0.9463 | | 1.0000 | |
| Fully vaccinated 9 months | | 0.0057 | | 0.0972 | | 0.9535 | | 1.0000 | |
| **Health Facility within 15 km** | | | | | | | | | |
| Number of ANC visits | | 0.0770 | | 0.4315 | | 0.8585 | | 1.0000 | |
| Four ANC visits | | 0.1422 | | 0.1259 | | 0.2603 | | 1.0000 | |
| ANC with professional | | -0.1101 | | 0.0665 | | 0.0998 | | 1.0000 | |
| Offered HIV test at ANC | | -0.0701 | | 0.0831 | | 0.4002 | | 1.0000 | |
| Tested for HIV at ANC | | -0.0208 | | 0.0788 | | 0.7920 | | 1.0000 | |
| Knowledge of HIV vertical transmission | | 0.0585 | | 0.0803 | | 0.4676 | | 1.0000 | |
| Knowl. of HIV vert. transm. prevention | | 0.0091 | | 0.0664 | | 0.8911 | | 1.0000 | |
| Institutional delivery | | 0.0324 | | 0.0755 | | 0.6682 | | 1.0000 | |
| Institutional delivery with professional | | 0.0221 | | 0.0766 | | 0.7732 | | 1.0000 | |
| Fully vaccinated 12 months | | -0.0880 | | 0.1010 | | 0.3848 | | 1.0000 | |
| Fully vaccinated 9 months | | -0.0156 | | 0.0862 | | 0.8566 | | 1.0000 | |
| **Referral health facility** | | | | | | | | | |
| Number of ANC visits | | 0.0146 | | 0.3308 | | 0.9649 | | 1.0000 | |
| Four ANC visits | | -0.0268 | | 0.0982 | | 0.7851 | | 1.0000 | |
| ANC with professional | | -0.0905 | | 0.0589 | | 0.1262 | | 0.6312 | |
| Offered HIV test at ANC | | 0.1586 | | 0.0678 | | 0.0205 | | 0.1643 | |
| Tested for HIV at ANC | | 0.2215 | | 0.0627 | | 0.0005 | | **0.0059** | |
| Knowledge of HIV vertical transmission | | 0.2518 | | 0.0936 | | 0.0079 | | **0.0788** | |
| Knowl. of HIV vert. transm. prevention | | 0.2054 | | 0.0801 | | 0.0112 | | 0.1011 | |
| Institutional delivery | | 0.1711 | | 0.0829 | | 0.0406 | | 0.2839 | |
| Institutional delivery with professional | | 0.1390 | | 0.0847 | | 0.1029 | | 0.6175 | |
| Fully vaccinated 12 months | | -0.0129 | | 0.1351 | | 0.9241 | | 1.0000 | |
| Fully vaccinated 9 months | | 0.0773 | | 0.0910 | | 0.3967 | | 1.0000 | |
|  | | | | | | | | | |
| \|  \| **Coefficient** \| **Standard Error** \| **p-Value** \| **Bonferroni-Holm p-value** \| \| --- \| --- \| --- \| --- \| --- \| | | | | | | | | | |
| **Effects of PBF by Exposure and Province (Gaza)** | | | | | | | | | |
| **Health Facility** | | | | | | | | | |
| Number of ANC visits | | 0.0710 | | 0.1940 | | 0.7146 | | 1.0000 | |
| Four ANC visits | | 0.0288 | | 0.0577 | | 0.6184 | | 1.0000 | |
| ANC with professional | | 0.0584 | | 0.0243 | | 0.0172 | | 0.1887 | |
| Offered HIV test at ANC | | 0.0626 | | 0.0578 | | 0.2799 | | 1.0000 | |
| Tested for HIV at ANC | | 0.0883 | | 0.0739 | | 0.2338 | | 1.0000 | |
| Knowledge of HIV vertical transmission | | 0.1300 | | 0.0898 | | 0.1492 | | 1.0000 | |
| Knowl. of HIV vert. transm. prevention | | 0.2630 | | 0.1100 | | 0.0175 | | 0.1887 | |
| Institutional delivery | | 0.0539 | | 0.0603 | | 0.3726 | | 1.0000 | |
| Institutional delivery with professional | | 0.0056 | | 0.0567 | | 0.9210 | | 1.0000 | |
| Fully vaccinated 12 months | | 0.0177 | | 0.0766 | | 0.8172 | | 1.0000 | |
| Fully vaccinated 9 months | | 0.0016 | | 0.0778 | | 0.9838 | | 1.0000 | |
| **Health Facility within 15 km** | | | | | | | | | |
| Number of ANC visits | | 0.2430 | | 0.1798 | | 0.1779 | | 0.9188 | |
| Four ANC visits | | 0.0318 | | 0.0579 | | 0.5837 | | 1.0000 | |
| ANC with professional | | 0.0387 | | 0.0216 | | 0.0749 | | 0.7488 | |
| Offered HIV test at ANC | | 0.0835 | | 0.0571 | | 0.1445 | | 0.9188 | |
| Tested for HIV at ANC | | 0.1056 | | 0.0667 | | 0.1149 | | 0.9188 | |
| Knowledge of HIV vertical transmission | | 0.0862 | | 0.0776 | | 0.2677 | | 1.0000 | |
| Knowl. of HIV vert. transm. prevention | | 0.2217 | | 0.0909 | | 0.0154 | | 0.1692 | |
| Institutional delivery | | 0.0899 | | 0.0519 | | 0.0844 | | 0.7596 | |
| Institutional delivery with professional | | 0.0774 | | 0.0503 | | 0.1248 | | 0.9188 | |
| Fully vaccinated 12 months | | 0.0406 | | 0.0746 | | 0.5871 | | 1.0000 | |
| Fully vaccinated 9 months | | 0.0079 | | 0.0870 | | 0.9280 | | 1.0000 | |
| **Referral health facility** | | | | | | | | | |
| Number of ANC visits | | 0.2012 | | 0.1839 | | 0.2749 | | 1.0000 | |
| Four ANC visits | | 0.0024 | | 0.0545 | | 0.9655 | | 1.0000 | |
| ANC with professional | | 0.0629 | | 0.0279 | | 0.0249 | | 0.2241 | |
| Offered HIV test at ANC | | 0.1141 | | 0.0606 | | 0.0608 | | 0.4258 | |
| Tested for HIV at ANC | | 0.2310 | | 0.0731 | | 0.0018 | | **0.0194** | |
| Knowledge of HIV vertical transmission | | 0.1074 | | 0.0768 | | 0.1629 | | 0.9774 | |
| Knowl. of HIV vert. transm. prevention | | 0.3070 | | 0.1010 | | 0.0026 | | **0.0262** | |
| Institutional delivery | | 0.1112 | | 0.0534 | | 0.0384 | | 0.3073 | |
| Institutional delivery with professional | | 0.0699 | | 0.0505 | | 0.1677 | | 0.9774 | |
| Fully vaccinated 12 months | | 0.0811 | | 0.0718 | | 0.2599 | | 1.0000 | |
| Fully vaccinated 9 months | | 0.0410 | | 0.0841 | | 0.6259 | | 1.0000 | |
